# Real-world multicenter evidence on Nefecon for attenuating proteinuria and kidney function decline in IgA nephropathy

**DOI:** 10.3389/fimmu.2026.1860166

**Published:** 2026-08-18

**Authors:** Shasha Chen, Lanping Jiang, Yanyan Zhang, Wei Wang, Qiong Wen, Naya Huang, Shaozhen Feng, Guisen Li, Wei Chen

**Affiliations:** 1 Department of Nephrology, Sichuan Academy of Medical Sciences & Sichuan Provincial People’s Hospital, Chengdu, China; 2 Department of Nephrology, The First Affiliated Hospital, Sun Yat-Sen University, Guangzhou, China; 3 National Health Commission (NHC) Key Laboratory of Clinical Nephrology (Sun Yat-Sen University) and Guangdong Provincial Key Laboratory of Nephrology, Guangzhou, China; 4 School of Medicine, University of Electronic Science and Technology of China, Chengdu, China

**Keywords:** IgA nephropathy, Nefecon, proteinuria and kidney function, delay response, real-world evidence, advanced kidney disease

## Abstract

**Introduction:**

Targeted-release budesonide (Nefecon) has demonstrated efficacy in IgA nephropathy (IgAN) trials, yet real-world data across disease severity spectrum remains limited.

**Methods:**

This multicenter, retrospective cohort study included 148 biopsy-proven IgAN adults (Nefecon =79; RASi =69). Primary endpoints were 9-month changes in proteinuria and eGFR. Mahalanobis distance matching within propensity score calipers addressed baseline imbalances. Response dynamics and safety were secondarily evaluated.

**Results:**

Despite worse baseline eGFR (median 54.2 vs. 85.3 mL/min/1.73m²; P < 0.001) and more severe pathological lesions in the Nefecon group compared to RASi group, Nefecon was associated with greater proteinuria reduction (-1.25 vs. –0.58 g/24h; P < 0.001) and favorable eGFR trajectories (+2.7 vs. -11.2 mL/min/1.73m²; between-group difference 13.9 mL/min/1.73m^2^; 95% CI 12.7 to 15.1; P <0.001), findings remained largely consistent after Mahalanobis distance matching adjustment. Complete and partial remission rates were higher (29.1% and 54.2% vs. 10.1% and 23.2%; P < 0.001), with 59.5% achieving ≥50% proteinuria reduction versus 21.7% (RR 2.70, 95% CI 1.62–4.51). Treatment effects varied by baseline disease severity. Enhanced antiproteinuric efficacy was observed in patients with proteinuria >1.5 g/24h (interaction P = 0.003), while kidney function preservation was most pronounced in those with eGFR <60 mL/min/1.73m². Notably, 51.1% of initial non-responders achieved remission by 9 months, with complete remission rates increasing 4.6-fold over time. Adverse events were consistent with corticosteroid exposure; menstrual disturbance occurred in 39.5% of females. Serious adverse events were rare (2.5%).

**Conclusion:**

This real-world observational analysis shows Nefecon associated with improved proteinuria and eGFR outcomes across IgAN severity, extending randomized trial evidence to patients with advanced CKD and revealing substantial delayed response.

## 1 Introduction

IgA nephropathy (IgAN) is the most common primary glomerulonephritis worldwide and a leading cause of kidney failure in young individuals (1, 2). Its prevalence is highest in the Asian Pacific region (1), accounting for approximately 39.73% of kidney biopsies in China (3). Over 30% of patients are diagnosed at chronic kidney disease (CKD) stage 3 or higher, and up to 60% progress to kidney failure within 15 years after diagnosis (4–6). Preventing progression to end-stage kidney disease (ESKD) is therefore a crucial therapeutic goal. Proteinuria and persistent hematuria are key prognostic indicators of kidney outcome in IgAN (4, 7, 8).

Increased understanding of IgAN pathogenesis, particularly the “four-hit hypothesis”, has spurred the development of novel target therapies (9). Several investigational agents have advanced substantially in clinical development, offering more treatment options for IgAN patients (10–17). Nefecon, a targeted-release formulation of budesonide, is the first approved therapy for IgAN (9). Nefecon acts locally on the gut-associated lymphoid tissue (GALT) of the distal ileum to reduce the production of pathogenic galactose-deficient IgA1 (Gd-IgA1) (18, 19). The Phase 3 NefIgArd trial demonstrated that a 9-month course of Nefecon significantly reduces urine protein-to-creatinine ratio (UPCR) and attenuates estimated glomerular filtration rate (eGFR) decline (11). These benefits were sustained over two years (9 months of treatment plus 15 months of follow-up) (10), with modeling predicting a delay in kidney failure by approximately 12.8 years (20).

Real-world evidence for Nefecon in Chinese patients remains limited. Two recent studies reported 40 and 60 Nefecon-treated patients, respectively, but both were single-center and uncontrolled (21, 22). Another single-center study of 11 patients examined those with severe renal impairment (eGFR <35 mL/min/1.73m²) (23). The phase 3 NefIgArd trial, a double-blind placebo-controlled study, restricted enrollment to eGFR 35–90 mL/min/1.73m² and included only 62 Chinese patients (9–11). Whether these findings apply to Chinese patients with more advanced disease, who typically present with lower eGFR, requires confirmation in a larger controlled cohort. In addition, the trajectory of individual patient response over the full 9-month course has not been characterized. We designed this multicenter real-world study with a RASi comparator to address these questions.

## 2 Materials and methods

### 2.1 Study design and participants

This multicenter, retrospective, observational study was conducted at two tertiary medical centers in China. Adult patients (≥18 years) with a biopsy-proven primary IgAN who completed at least 9 months of follow-up were enrolled between January 2024 and March 2025. The key inclusion criteria was baseline eGFR ≥25 mL/min/1.73m². All eligible patients received either Nefecon or supportive care alone. Exclusion criteria were secondary IgAN, rapidly progressive glomerulonephritis or other significant kidney diseases, non-adherence to regular follow-up, and concurrent use of telitacicept or systemic corticosteroids. The study protocol was approved by the Institutional Review Board of all participating centers (Ethics Committee of Sichuan Provincial People’s Hospital, Approval No.2024686; The First Affiliated Hospital of Sun Yat-Sen University, Approval No. [2021]608). The requirement for informed consent was waived due to the retrospective nature of the study. A STROBE-compliant participant flow diagram is provided in **Supplementary Figure 1**.

### 2.2 Treatment and data collection

Eligible patients were categorized into two groups: the Nefecon group received Nefecon (16 mg once daily) for 9 months (77.7% as first-line therapy vs. 22.3% as add-on after ≥3 months of optimized supporting care), and the RASi (renin-angiotensin system inhibitor) group (including angiotensin-converting enzyme inhibitor (ACEi) or angiotensin II receptor blocker (ARB)). Sodium-glucose cotransporter-2 inhibitor (SGLT2-i) was also permitted in both groups. Data on demographics, clinical characteristics, laboratory parameters (including 24-hour proteinuria and eGFR), and concomitant medications were systematically extracted from electronic medical records at baseline, 3, 6, and 9 months. The time from renal biopsy to treatment initiation was recorded for all patients and included as a covariate in subgroup analyses. Adverse events (AEs) and serious adverse events (SAEs) were adjudicated through comprehensive review of all clinical records by GCP-certified investigators, supplemented by telephone follow-ups when needed. AEs related to Nefecon and those previously reported in studies were included (10, 11, 23). SAEs were defined as events leading to death, a life-threatening experience, inpatient hospitalization, or persistent significant disability.

### 2.3 Outcomes

Primary efficacy endpoints were absolute change in 24-hour proteinuria and eGFR from baseline to 9 months. Secondary endpoints included rates of complete clinical remission (CR, proteinuria <0.3 g/24h), partial remission (PR, proteinuria <1.0 g/24h or a ≥50% reduction from baseline), and non-response (NR, failed to achieve CR or PR), as well as the proportion of patients achieving ≥30% and ≥50% proteinuria reduction from baseline.

### 2.4 Statistical analysis

All statistical analyses were performed using SPSS Statistics version 26.0 (IBM Corp.). Continuous variables were presented as mean ± standard deviation (normal distribution) or median with interquartile range (skewed distribution). Categorical data were expressed as percentages. Group comparisons used independent samples t-test, Mann-Whitney U test, Chi-square, or Fisher’s exact test, as appropriate. To assess consistency of the treatment effect, pre-specified subgroup analyses were performed based on baseline demographics and disease characteristics. Interaction terms were included in the models to formally test for effect modification. A two-sided *P* < 0.05 was considered significant. The Holm-Bonferroni correction was applied for multiple comparisons of response rates across time points.

To minimize confounding by indication, we employed Mahalanobis distance matching within propensity score calipers as a variant of propensity score matching to balance the two groups. The propensity score was estimated using multivariable logistic regression incorporating the following baseline variables, selected based on their clinical relevance and potential association with both treatment assignment and kidney outcomes: age, sex, 24-hour proteinuria, eGFR, serum albumin, SGLT2i use, and Oxford MEST-C classification scores. Matching was performed using a 1:1 nearest-neighbor algorithm without replacement. For each Nefecon-treated patient, we first identified RASi-treated patients whose propensity scores fell within a caliper of 0.2 standard deviations of the logit-transformed propensity score. Among these candidates, we selected the patient with the smallest Mahalanobis distance based on the matching covariates. This hybrid approach ensures that matched pairs fall within the region of common support while directly optimizing multivariate covariate similarity (24, 25). Covariate balance before and after matching was assessed using standardized mean differences (SMDs). An absolute SMD < 0.1 was considered indicative of negligible imbalance. Balance was visualized using a Love plot.

As a sensitivity analysis, we compared Mahalanobis matching with Stabilized Inverse Probability of Treatment Weighting (Stabilized IPTW). Mahalanobis matching achieved superior covariate balance (mean |SMD| = 0.091; 12/12 covariates with |SMD| < 0.2) compared to Stabilized IPTW (mean |SMD| = 0.216; 6/12 covariates with |SMD| < 0.2). Therefore, Mahalanobis matching was selected as the primary analytical approach.

### 2.5 Missing data handling

Missing data for baseline covariates and longitudinal follow-up variables were handled using multiple imputation by chained equations (MICE). For key efficacy endpoints (24-hour urine proteinuria and eGFR), missing values were imputed using predictive mean matching, incorporating baseline characteristics, concomitant medications, and prior visit values as predictors. Time-point-specific missing data rates are reported in **Supplementary Table 2**. Overall, missing data for baseline covariates were <10%, consistent with the high data capture standards of our tertiary care centers’ standardized electronic medical record (EMR) systems, where 24-hour urine collection is mandated as a core follow-up parameter for all IgAN patients.

## 3 Results

### 3.1 Study population and baseline characteristics

Before matching, the study cohort comprised 148 patients (69 treated with RASi and 79 treated with Nefecon). Baseline characteristics differed between groups (**Table 1**). Patients in the Nefecon group had significantly lower eGFR (median 54.2 vs. 85.3 mL/min/1.73m²), lower serum albumin (median 41.5 vs. 43.2 g/L), and less frequent SGLT2i use (48.1% vs. 69.6%). They also had more severe histologic lesions, with higher proportions of segmental glomerulosclerosis (S1: 74.7% vs. 56.5%), tubular atrophy/interstitial fibrosis (T: 44.3% vs. 17.4%), and crescents (C: 38% vs. 10.1%). Concomitant immunosuppressants were used only in the Nefecon group (hydroxychloroquine 20.3%, mycophenolate mofetil 8.9%, cyclophosphamide 2.5%).

**Table 1:** Baseline characteristics of patients with IgAN.

Variables	Total (n = 148)	RASi group (n = 69)	Nefecon group (n = 79)	P
Age, years	36.9 ± 10.0	36.7 ± 9.7	37.0 ± 10.2	0.893
Female sex, n (%)	79 (53.4)	41 (59.4)	38 (48.1)	0.226
Total follow-up duration from study enrollment, months	25.0 (11.8, 67.2)	39.0 (12.0, 72.0)	17.0 (11.0, 49.0)	0.056
BMI, Kg/m²	23.0 ± 4.2	22.3 ± 4.2	23.7 ± 4.1	0.147
SBP, mmHg	129.6 ± 24.7	125.9 ± 26.7	133.2 ± 22.1	0.163
DBP, mmHg	83.8 ± 14.5	83.0 ± 12.4	84.7 ± 16.3	0.375
Hypertension, n (%)	35 (23.7)	20 (29.0)	15 (19.0)	0.489
DM, n (%)	3 (2.0)	1 (1.4)	2 (2.5)	1.000
Proteinuria, g/24h	2.0 ± 1.6	1.7 ± 0.6	2.2 ± 2.1	0.949
SCr, μmol/L	110.0 (76.5, 142.0)	95.1 (63.0, 111.0)	127.5 (104.4, 163.1)	< 0.001
eGFR, ml/min/1.73m²	64.8 (46.2, 95.0)	85.3 (56.4, 113.4)	54.2 (40.0, 78.1)	< 0.001
UA, μmo/L	390.5 ± 118.8	372.9 ± 117.0	407.3 ± 118.9	0.119
Serum albumin, g/L	42.1 (39.0, 45.0)	43.2 (40.6, 45.5)	41.5 (36.5, 43.0)	< 0.001
Hematuria, RBC/μL	86.0 (22.0, 325.7)	86.0 (20.0, 396.3)	86.0 (31.0, 248.0)	0.762
RASi, n (%)	136 (91.9)	69 (100)	67 (84.8)	0.001
SGLT2i, n (%)	86 (58.1)	48 (69.6)	38 (48.1)	0.013
Concomitant immunosuppressant use				
Steroids, n (%)	0	0	0	–
Cyclophosphamide, n (%)	2 (1.4)	0	2 (2.5)	–
Mycophenolate mofetil (MMF), n (%)	7 (4.7)	0	7 (8.9)	–
Hydroxychloroquine (HCQ), n (%)	16 (10.8)	0	16 (20.3)	–
MEST-C score, n (%)				
M1	79 (53.3)	39 (56.5)	40 (50.6)	0.611
E1	40 (27.0)	20 (29.0)	20 (25.3)	0.938
S1	98 (66.2)	39 (56.5)	59 (74.7)	0.004
T1	34 (23.0)	9 (13.0)	25 (31.6)	< 0.001
T2	13 (8.8)	3 (4.3)	10 (12.7)	0.075
C1	36 (24.3)	7 (10.1)	29 (36.7)	< 0.001
C2	1 (0.7)	0	1 (1.3)	

BMI, body mass index; DM, diabetes mellitus; SBP, Systolic Blood Pressure; DBP, Diastolic Blood Pressure; SCr, Serum creatinine; eGFR, estimated glomerular filtration rate; UA, uric acid; HCQ, Hydroxychloroquine, SGLT2i, Sodium-Glucose Cotransporter 2 Inhibitor. M, mesangial hypercellularity (M1, >50% of glomeruli show mesangial hypercellularity); E, endocapillary hypercellularity (E1, any glomeruli show endocapillary hypercellularity); S, segmental glomerulosclerosis (S1, present in any glomeruli); T, tubular atrophy/interstitial fibrosis (T1, 26%-50% of cortical area; T2, >50% of cortical area); C, crescents (C1, 1%-25% of glomeruli; C2, ≥25% of glomeruli). eGFR was calculated using the Chronic Kidney Disease Epidemiology Collaboration (CKD-EPI) 2009 equation for adults.

After Mahalanobis distance matching within propensity score calipers, 40 matched pairs (n=80) were retained (**Supplementary Table 1**). Among the matching covariates, most had absolute SMDs below 0.1 (**Supplementary Table 1**, **Supplementary Figure 2**). The SMD for eGFR decreased from 0.886 before matching to 0.077 after matching; serum albumin from 0.619 to 0.088; proteinuria from 0.343 to 0.009; SGLT2i use from 0.435 to 0.100; and Oxford MEST-C components all achieved SMDs < 0.2 (M: 0.156; E: 0.057; S: 0.055; T1: 0.057; C1: 0.125). No statistically significant differences were observed between matched groups for any baseline variable (all *P* > 0.05).

### 3.2 Primary efficacy endpoints

**Figure 1:**
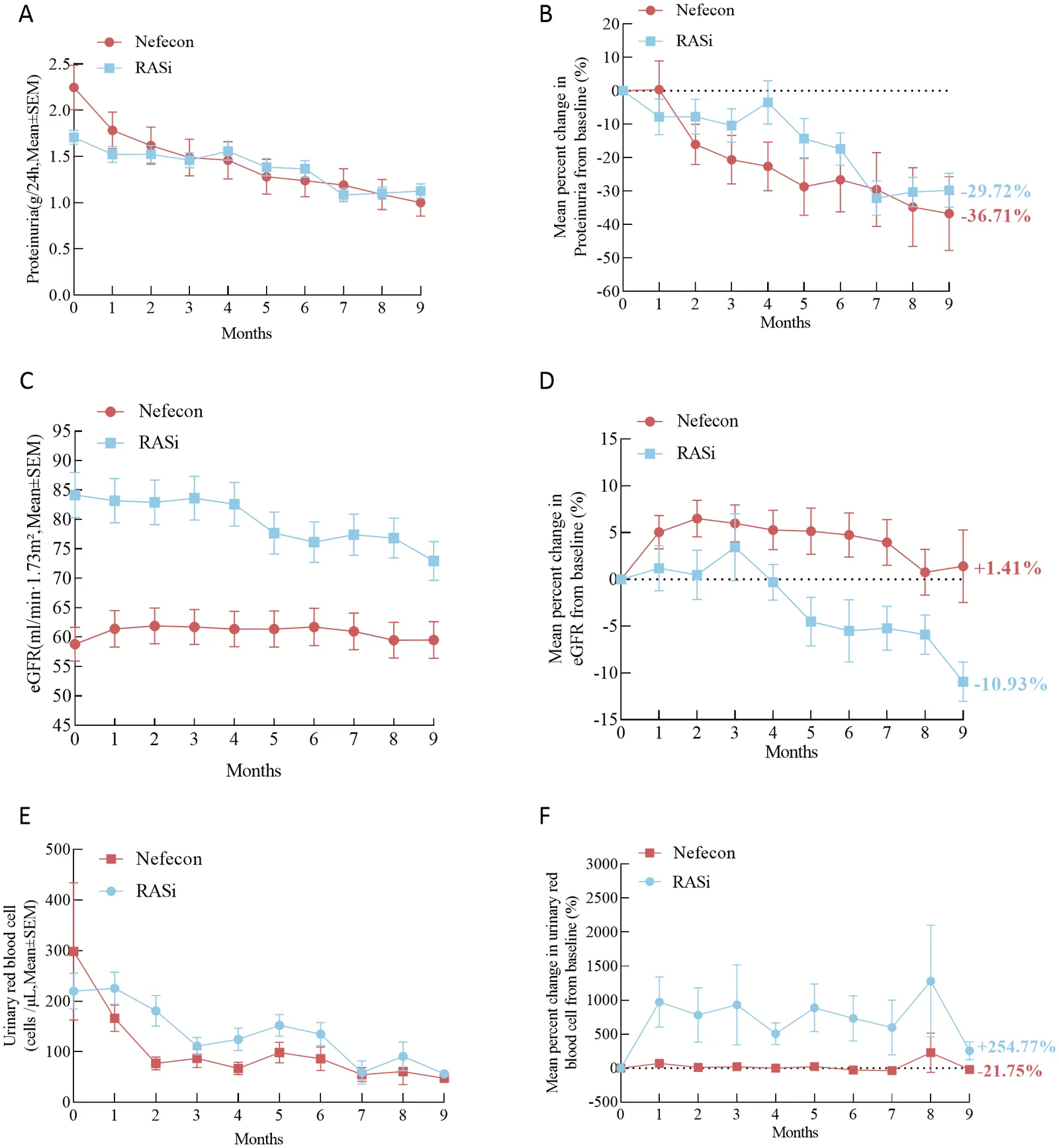
Changes in clinical parameters from baseline to month 9. **(A)** Mean change in proteinuria **(B)** Percent change in proteinuria from baseline; **(C)** Mean change in eGFR; **(D)** Percent change in eGFR from baseline; **(E)** Mean change in hematuria (urine red blood cell count/μL); **(F)** Percent change in hematuria from baseline. Data are presented as mean ± SEM. Red lines represent the Nefecon group (n=79); blue lines represent the RASi group (n=69). eGFR, Estimated Glomerular Filtration Rate.

After 9 months of treatment, Nefecon treatment resulted in a significantly greater proteinuria reduction compared to RASi (**Figures 1A, B**). The mean absolute reduction in 24-hour proteinuria was significantly greater with Nefecon (-1.25±0.20 g) than with RASi (-0.58±0.09 g, P < 0.001), corresponding to relative reductions of -36.71% and -29.72%, respectively (treatment-time interaction P < 0.001).

When analyzing relative changes in eGFR, the Nefecon group showed a mean increase of 1.41% from baseline, which was significantly better than the 10.93% decline observed in the RASi group. The mean eGFR change was +2.7 ± 3.4 mL/min/1.73 m² with Nefecon, compared to a decline of -11.2 ± 3.7 mL/min/1.73 m² with RASi, resulting in a between-group difference of 13.9 mL/min/1.73 m² (95% CI 12.7 to 15.1; P<0.001) (**Figures 1C, D**). Analyses of both absolute and percent changes consistently favored Nefecon for all endpoints.

In addition, Nefecon treatment was associated with a significant reduction in urinary red blood cells compared to RASi (**Figures 1E, F**). The Nefecon group showed a 21.75% decrease from baseline in urinary red blood cell count, while the RASi group demonstrated a 254.77% increase.

To assess the robustness of the primary findings, we repeated the analyses in the Mahalanobis- matched cohort (n=80). In this matched cohort, the mean eGFR change was +0.62 ± 9.80 mL/min/1.73m² (median -0.70 [IQR: -6.22 to 7.50]) in the Nefecon group and -8.42 ± 15.72 mL/min/1.73m² (median -3.90 [IQR: -15.93 to 0.50]) in the RASi group (Mann-Whitney U P = 0.008; **Supplementary Figure 3**), a between-group difference of 9.05 mL/min/1.73m². In addition, 47.5% of Nefecon-treated patients showed eGFR improvement versus 27.5% in the RASi group. For proteinuria, both groups had significant reductions from baseline (Nefecon: Wilcoxon signed-rank P<0.001; RASi: P = 0.001). The mean proteinuria reduction was -0.71 ± 1.09 g/24h in the Nefecon group versus -0.48 ± 0.84 g/24h in the RASi group.

We also evaluated baseline serum IgA as a predictor of Nefecon response. Spearman correlation analysis showed a weak, non-significant positive association between baseline IgA and 9-month proteinuria reduction (r = 0.124, P = 0.355, n = 58). Linear regression confirmed limited predictive value (R²= 0.036), with baseline IgA explaining only 3.6% of response variance. When stratified by treatment response, baseline IgA levels were comparable between responders and non-responders (3.12 ± 0.89 vs. 2.61 ± 0.78 g/L, P = 0.102). These findings, presented in **Supplementary Figure 7**, suggest baseline serum IgA is not a robust predictive biomarker for Nefecon response in this cohort.

### 3.3 Secondary efficacy endpoints and clinical remission

**Figure 2:**
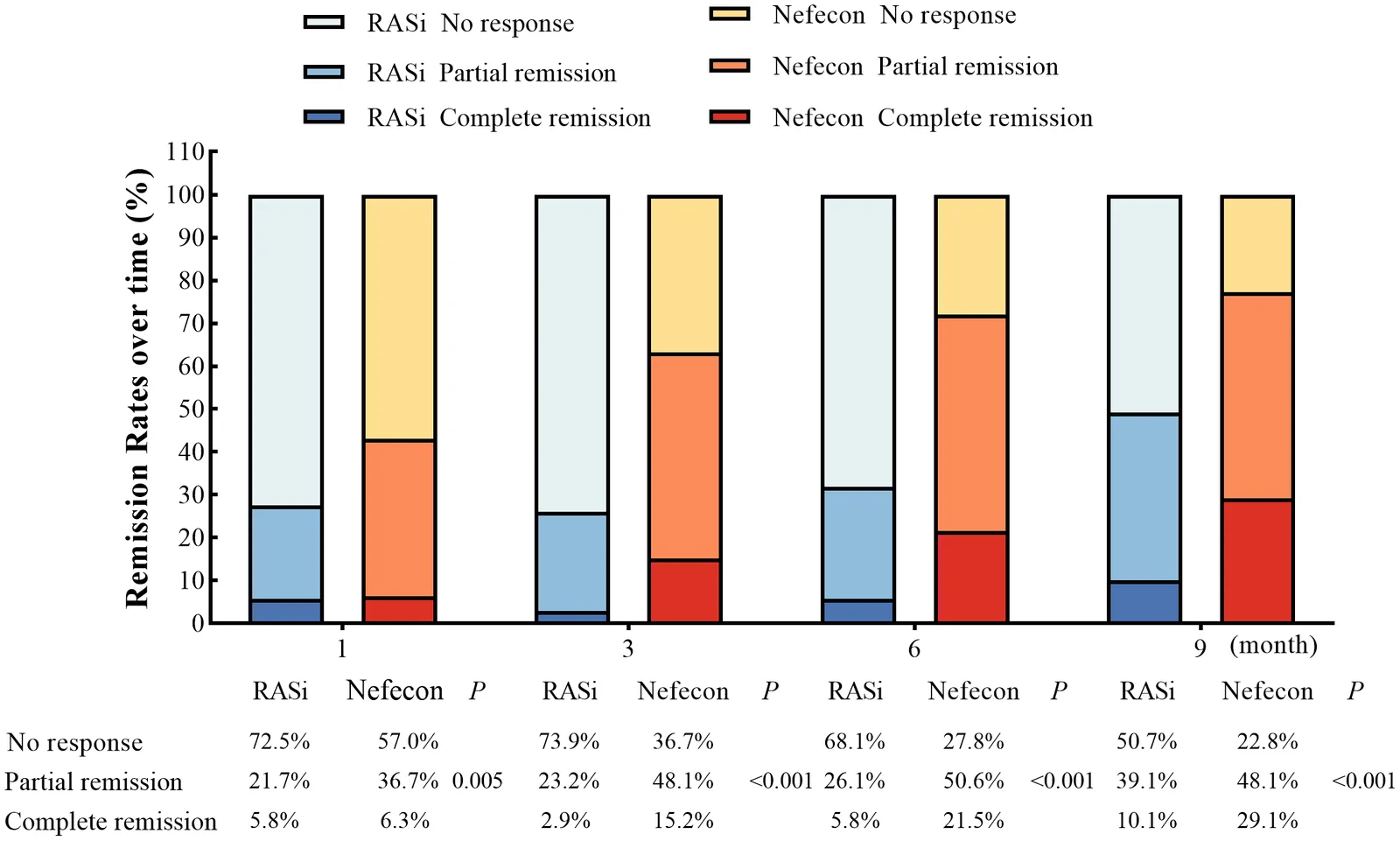
Comparison of remission rates between treatment groups over time. Stacked bars show the proportion of patients achieving complete remission, partial remission, or no response at 1, 3, 6, and 9 months. The Nefecon group demonstrated significantly higher cumulative remission rates (CR+PR) compared to the RASi group.

Longitudinal treatment responses are detailed in **Figure 2**. In the Nefecon group, the CR rate increased from 15.2% at 3 months to 29.1% at 9 months, compared with 2.9% to 10.1% in the RASi group (log-rank *P* < 0.001). PR rates were significantly higher in the Nefecon group at 3, 6, and 9 months (all *P* < 0.01), and the proportion of non-responders was lower compared to RASi group (**Figure 2**).

**Figure 3:**
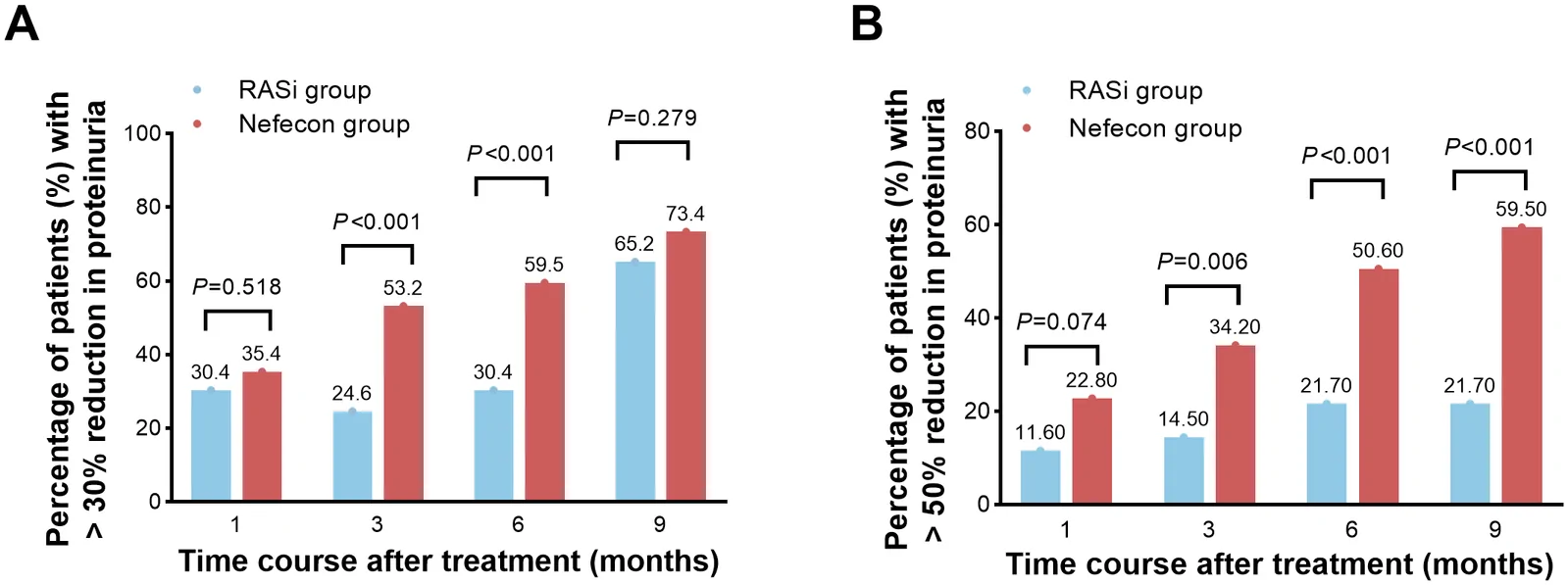
Proteinuria response rates according to predefined thresholds. Proportion of patients achieving **(A)** ≥30% and **(B)** ≥50% reduction in 24-hour proteinuria from baseline at 1, 3,6 and 9 months after treatment.

Threshold-based proteinuria reduction is shown in **Figure 3**. At 9 months, the proportion achieving ≥30% reduction was 73.4% in the Nefecon group versus 65.2% in the RASi group (RR 1.14, 95% CI 0.90-1.44), and the proportion achieving ≥50% reduction was 59.5% versus 21.7% (RR 2.7, 95% CI 1.62-4.51). The response difference between groups was evident from month 3 onward (*P* < 0.001).

In the matched cohort, the overall response rate (CR + PR) was 75% in the Nefecon group and 52.5% in the RASi group (**Supplementary Figure 4**); ≥50% proteinuria reduction was achieved in 47.5% versus 22.5% (**Supplementary Figure 5**). The matched analysis had reduced statistical power due to the 46% reduction in sample size. The non-significant P value for the proteinuria comparison in this subgroup should be interpreted with caution.

### 3.4 Subgroup analysis of treatment response

**Figure 4:**
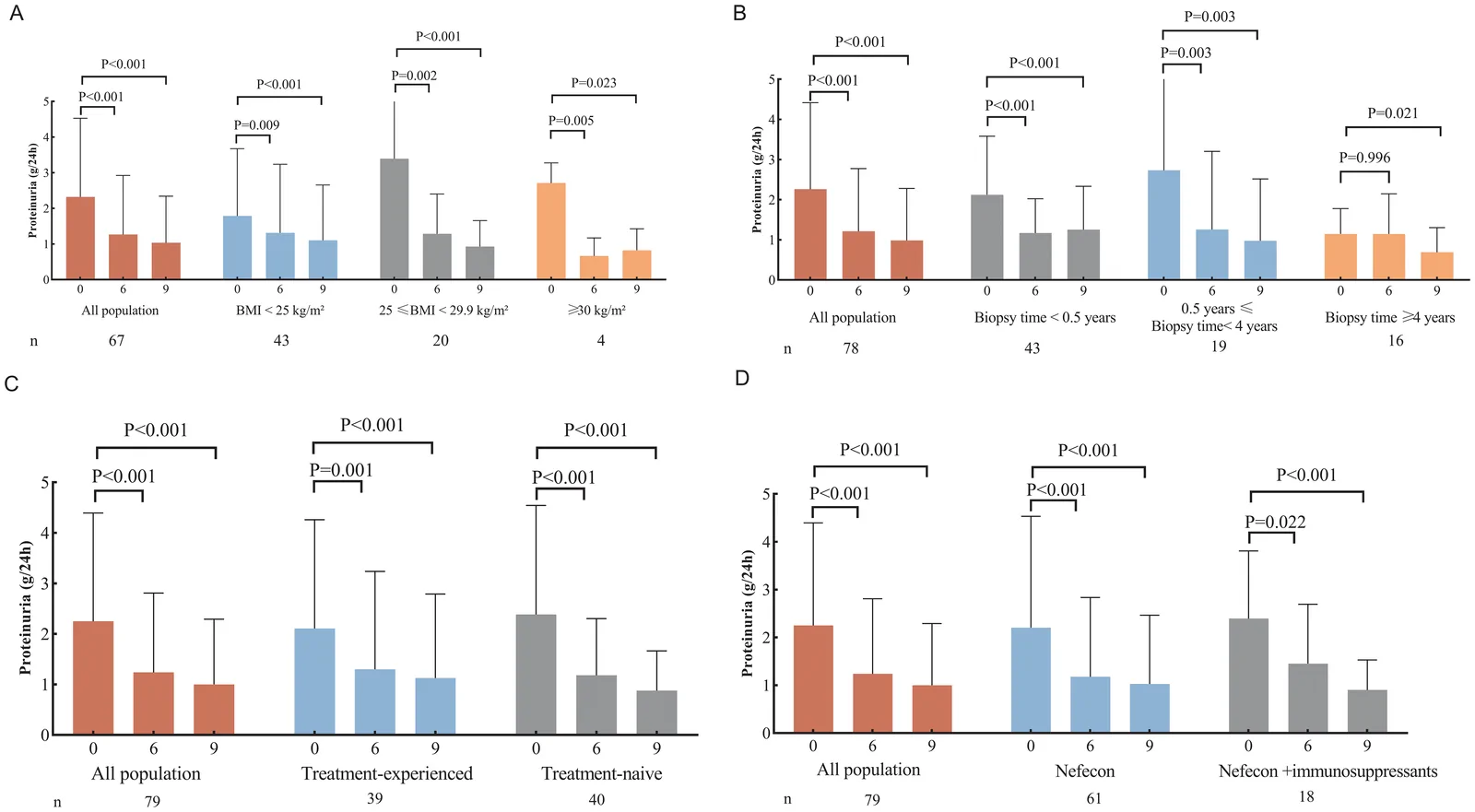
Consistency of proteinuria reduction with Nefecon across patient subgroups. Grouped bar chart depicting mean change in proteinuria from baseline to 9 months in the Nefecon group, stratified by **(A)** baseline BMI, **(B)** biopsy time, **(C)** Treatment-naive vs. treatment-experienced status, and **(D)** Nefecon monotherapy vs. Nefecon plus immunosuppressants. BMI categories: <25 kg/m², 25–29.9 kg/m², and ≥30 kg/m². Note, Treatment-naive group, patients who had never received any prior systemic therapy (including corticosteroids, immunosuppressants, or biologics) before study entry; Treatment-experienced group, patients who had previously received one or more systemic therapies for their underlying disease, regardless of duration or response. Nefecon monotherapy group, patients treated with Nefecon alone during the study period, without concomitant immunosuppressive agents; Nefecon plus immunosuppressants group, patients who received Nefecon in combination with any systemic immunosuppressive medication (e.g., azathioprine, mycophenolate, calcineurin inhibitors) during the study period.

Subgroup analyses demonstrated that the effect of Nefecon on proteinuria reduction was consistent across most pre-specified patient subgroups. The mean change in 24-hour proteinuria showed no significant interaction with baseline BMI categories (<25, 25–29.9, ≥30 kg/m²; *P* = 0.88), prior treatment history (*P >*0.10), or concomitant immunosuppressant use (*P >*0.10) (**Figures 4A, C, D**), as well as time from biopsy to treatment initiation (<0.5, 0.5–4, ≥4 years; median 2.0 months [IQR 0.1–23.0]; *P* = 0.79). However, the timing of the initial response varied. Among patients with an interval < 4 years, a significant proteinuria reduction emerged as early as 6 months, whereas in those with an interval > 4 years, a notable decrease was not observed until 9 months (**Figure 4B**). Results were consistent in the matched cohort (**Supplementary Figure 6**). As shown in **Supplementary Figure 8**, Nefecon was associated with significant proteinuria reduction from baseline to Month 9 across all pathological subgroups, including active lesions such as endocapillary hypercellularity (E1) and crescents (C1-2). Interaction tests for all MEST-C components were non-significant (all P>0.05).

**Figure 5:**
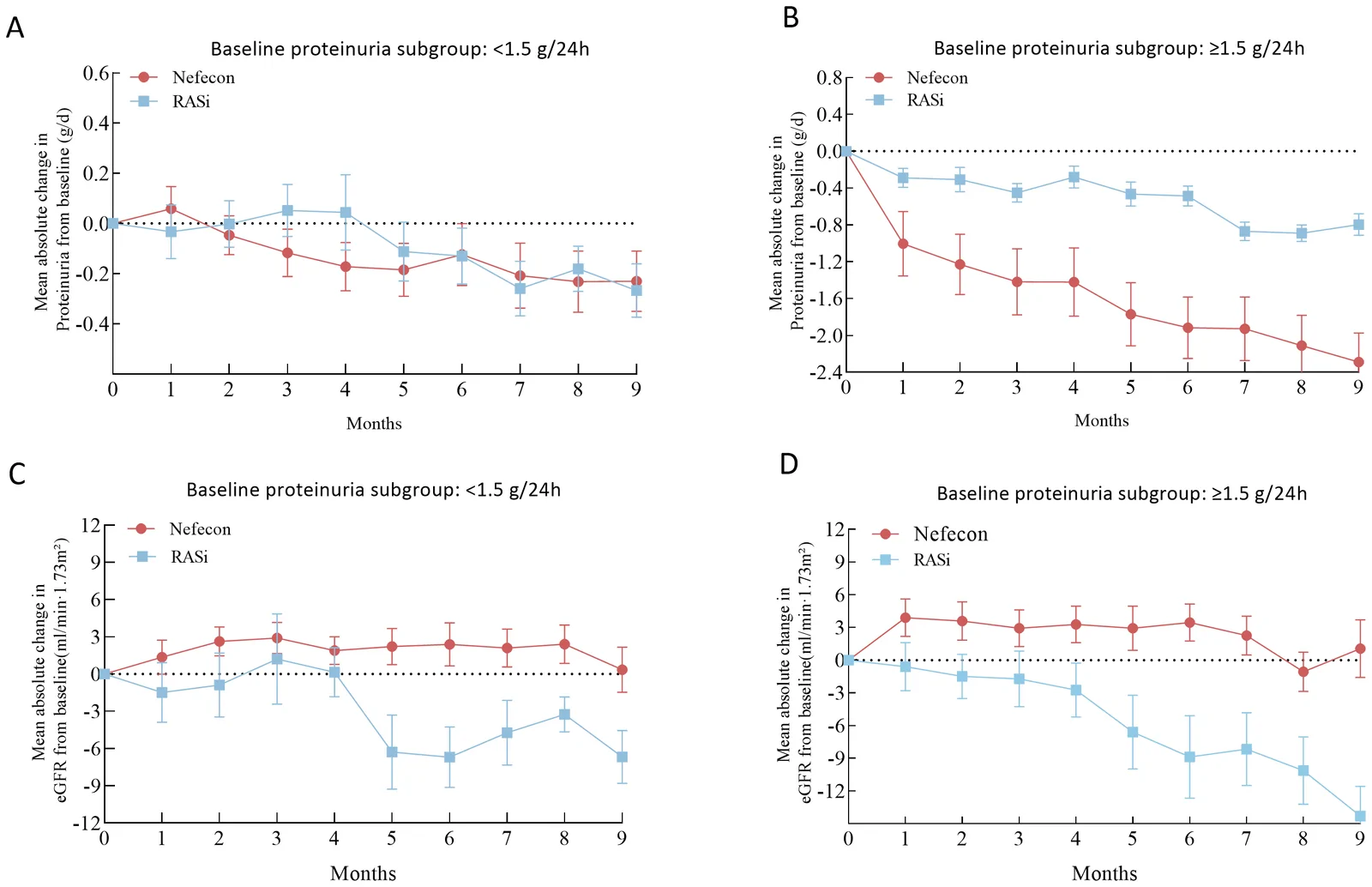
Treatment response of Nefecon stratified by baseline proteinuria. **(A)** Mean absolute change in proteinuria (g/24h) from baseline to Month 9 in patients with baseline proteinuria <1.5 g/24h (Nefecon, n=40; RASi, n=28). **(B)** Mean absolute change in proteinuria from baseline in patients with baseline proteinuria ≥1.5 g/24h (Nefecon, n=39; RASi, n=41). **(C)** Mean absolute change in eGFR (mL/min/1.73m²) from baseline in patients with baseline proteinuria <1.5 g/24h. Nefecon, n=40; RASi, n=28. **(D)** Mean absolute change in eGFR from baseline in patients with baseline proteinuria ≥1.5 g/24h. Nefecon, n=39; RASi, n=41. Data are presented as mean ± SEM.

The treatment effects differed by baseline disease severity (**Figure 5**). Among patients with baseline proteinuria >1.5 g/24h, the absolute proteinuria reduction was greater with Nefecon than with RASi (mean between-group difference: approximately -1.5 g/24h at 9 months; interaction P = 0.003, **Figure 5B**). In this subgroup, mean eGFR increased by approximately +1.1 mL/min/1.73 m² with Nefecon versus a decline of approximately -14.3 mL/min/1.73 m² with RASi group at 9 months (**Figure 5D**). In patients with baseline proteinuria <1.5 g/24h, the proteinuria reduction was smaller, though eGFR preservation remained evident (**Figures 5A, C**).

**Figure 6:**
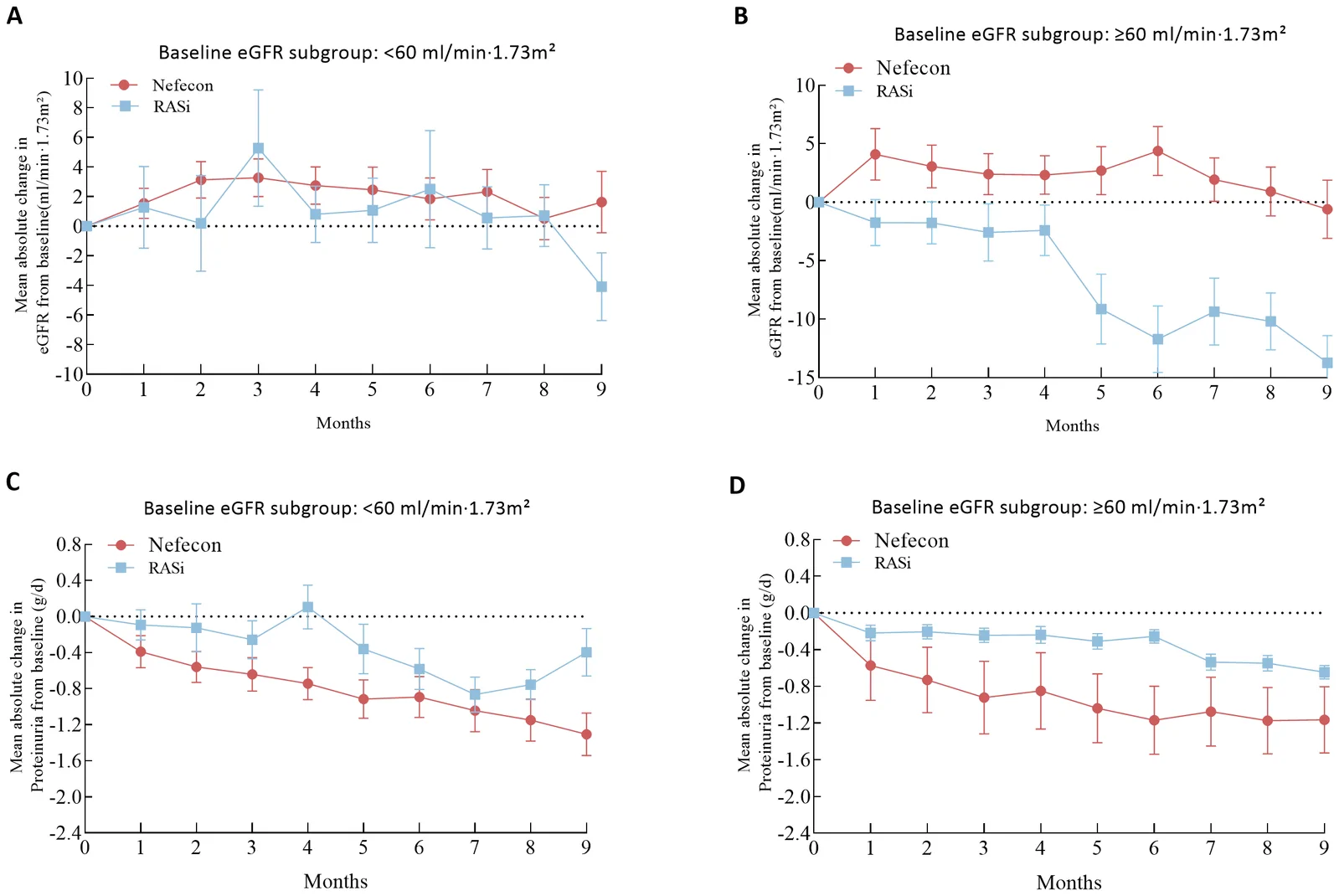
Treatment response of Nefecon stratified by baseline eGFR. **(A)** Mean absolute change in eGFR (mL/min/1.73m²) from baseline to Month 9 in patients with baseline eGFR <60 mL/min/1.73m² (Nefecon, n=46; RASi, n=18). **(B)** Mean absolute change in eGFR in patients with baseline eGFR ≥60 mL/min/1.73m² (Nefecon, n=33; RASi, n=51). **(C)** Mean absolute change in proteinuria (g/24h) from baseline in patients with baseline eGFR <60 mL/min/1.73m². Nefecon, n=46; RASi, n=18. **(D)** Mean absolute change in proteinuria in patients with baseline eGFR ≥60 mL/min/1.73m². Nefecon, n=33; RASi, n=51. Data are presented as mean ± SEM.

**Figure 6** shows the results by baseline eGFR. In patients with eGFR <60 mL/min/1.73 m², mean eGFR increase of approximately +2.0 mL/min/1.73 m² with Nefecon compared to a decline of approximately -4.0 mL/min/1.73 m² with RASi at 9 months (between-group difference: ~6.0 mL/min/1.73 m²; **Figure 6A**). In patients with eGFR ≥60 mL/min/1.73 m², mean eGFR change was approximately -0.6 mL/min/1.73 m^2^ with Nefecon versus -13.7 mL/min/1.73 m² with RASi (between-group difference: ~13.1 mL/min/1.73 m²; **Figure 6B**). At 9 months, the Nefecon group demonstrated a greater reduction in proteinuria than the RASi group in both baseline eGFR strata (<60 and ≥60 mL/min/1.73 m², **Figures 6C, D**).

### 3.5 Treatment response dynamics and temporal evolution

**Figure 7:**
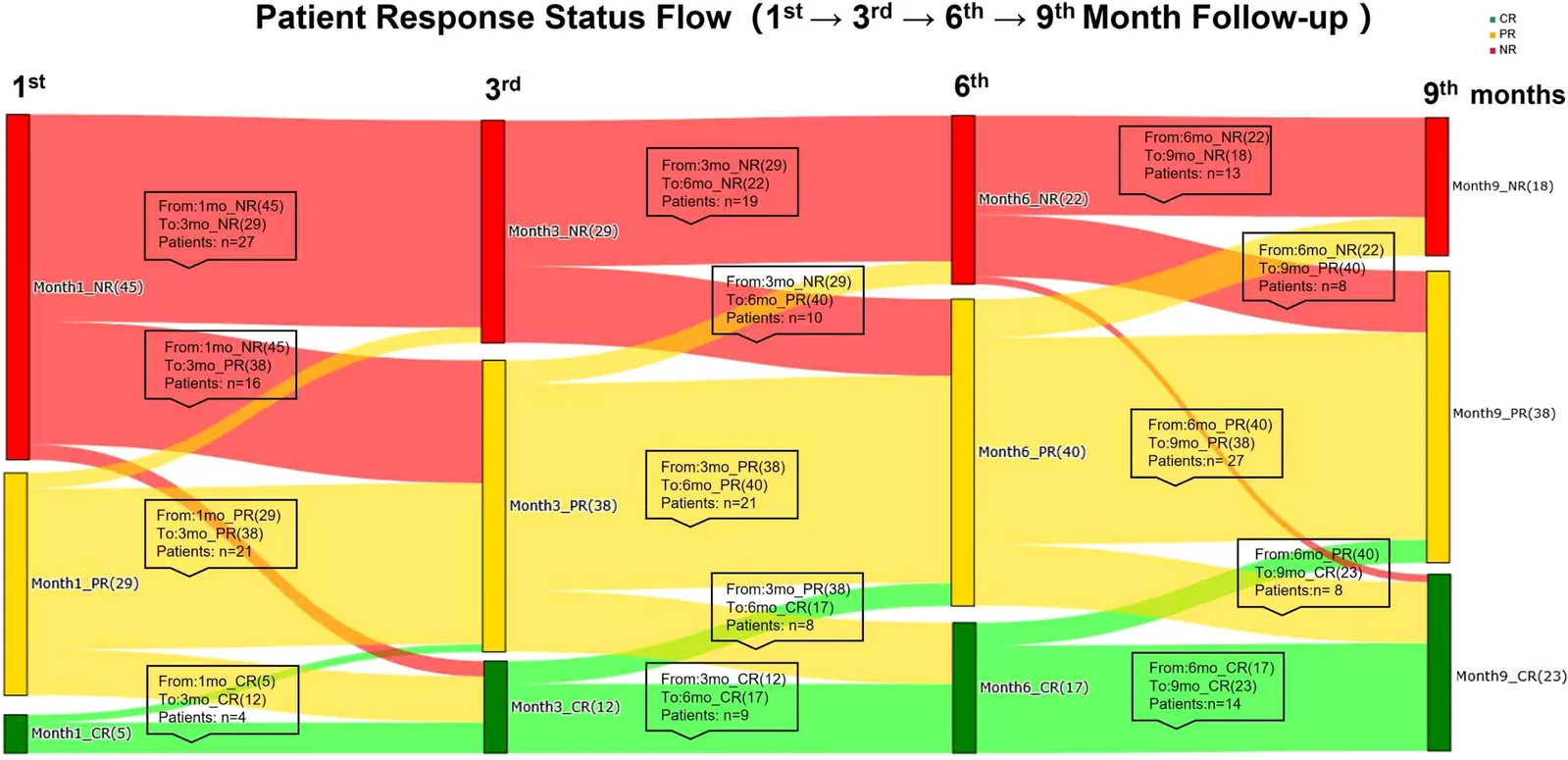
Dynamics of treatment response in the Nefecon group over 9 months. Sankey diagram illustrating the transition of individual patient response status at 1, 3, 6, and 9 months during Nefecon treatment. Each flow represents the trajectory of a patient’s response category over time. Node height reflects the proportion of patients in each response category at each time point. Numbers in boxes represent the sample size (n) for each subgroup. CR, complete remission; PR, partial remission; NR, no response. Among patients who were non-responders at Month 1 (n=45) and Month 3 (n=29), a substantial proportion achieved partial response or complete response by Month 9, demonstrating the dynamic and cumulative nature of treatment benefit with Nefecon. Response criteria: CR (proteinuria <0.3 g/24h); PR (proteinuria <1.0 g/24h or ≥50% reduction from baseline).

Analysis of longitudinal response trajectories revealed a substantial proportion of delayed treatment responders, challenging the paradigm that early non-response predicts ultimate treatment failure. In the Nefecon group (n=79), 45 patients (57.0%) were classified as non-responders (NR) at month 1. The number of NR decreased to 29 (36.7%) at month 3, 22 (27.8%) at month 6, and 18 (22.8%) at month 9. Notably, 51.1% (23/45) of initial non-responders ultimately achieved remission (PR or CR) by 9 months with 8 patients (17.8%) attaining CR despite initial NR status (**Figure 7**).

Conversely, early response demonstrated high durability and progressive enhancement. Among the 29 patients achieving PR at 1 month, 21 (72.4%) maintained PR status at 3 months, with 27 (93.1%) ultimately achieving PR or CR by 9 months. Notably, response deepening was observed over time, with the CR population expanding from 5 at month 1 to 12 at month 3, 17 at month 6, and 23 at month 9, representing a 4.6-fold increase and comprising 29.1% of the total evaluable population. Only 2 patients (4.4%) initially classified as NR at 1 month remained non-responders throughout the 9-month follow-up.

### 3.6 Treatment adherence and dose modifications

All 79 patients in the Nefecon group completed the 9-month course (**Supplementary Table 3**). In the Nefecon group, 4 patients (5.1%) required temporary dose reduction from 16 mg to 8 mg daily: two due to adverse events (menstrual disturbance, n = 1; acne and facial edema, n = 1), one due to patient decision, and one due to economic reasons. Dose reduction occurred in months 1-3 (n=1), months 4-6 (n=1), and months 7-9 (n = 2).

### 3.7 Safety and adverse events related to Nefecon

A complete summary of adverse events is presented in **Table 2**. During the 9-month treatment period, SAEs occurred in 2 of 79 patients (2.5%) in the Nefecon group; both required hospitalization (severe upper respiratory infection and hypertension). No deaths or life-threatening events occurred. The most common adverse events were consistent with systemic corticosteroid exposure. Among females of childbearing potential (n=38), menstrual disorder was reported in 15 (39.5%). Other frequent AEs included acne (15/79, 19.0%), moon facies (9/79, 11.4%), and facial edema (9/79, 11.4%). Peripheral edema occurred in 5 patients (6.3%), and upper respiratory infection in 7 (8.9%). New-onset hypertension among patients without baseline hypertension (n=19) was observed in 1 patient (1.3% of the overall cohort). Serial systolic and diastolic blood pressure remained stable throughout the 9-month treatment period in Nefecon group (**Supplementary Figure 9**).

**Table 2:** Adverse events in the Nefecon group (n=79).

Adverse event	n (%)
SAEs	2 (2.5)
Menstrual disorder (all females of childbearing age, n=38)	15 (39.5)
Acne	15 (19.0)
Moon face	9 (11.4)
Peripheral edema	5 (6.3)
Upper respiratory infections	7 (8.9)
Facial hirsutism	4 (5.1)
Weight gain	4 (5.1)
Insomnia	4 (5.1)
Fatigue	2 (2.5)
Rash	3 (3.8)
Muscle cramps	2 (2.5)
Facial edema	9 (11.4)
Hypertension	1 (1.3)
Incident hypertension (in patients without baseline hypertension, n=19)	1 (5.3)

SAE, Serious Adverse Event.

Menstrual disorder, also known as abnormal uterine bleeding, in patients of reproductive age is operationally defined as a significant deviation from the patient’s own regular menstrual pattern in cycle frequency, regularity, duration, or volume of flow.

## 4 Discussion

This multicenter real-world study extends the phase 3 NefIgArd trial findings to a broader, more advanced IgAN population (10, 11). Our cohort differs from the NefIgArd population in four clinically important dimensions: (i) broader eGFR range (median 54.2 mL/min/1.73m² in Nefecon group, with inclusion down to 25 mL/min/1.73m², vs. eGFR 35–90 mL/min/1.73m²in NefIgArd); (ii) more severe histologic lesions (T1/T2 in 44.3% and C1/C2 in 38.0% vs. approximately 25% and 15% in NefIgArd); (iii) inclusion of treatment-experienced patients with real-world adherence patterns (5.1% dose reduction); and (iv) Chinese population representation, addressing an ethnic-specific evidence gap given the highest IgAN prevalence in the Asia-Pacific region. Rather than replicating the NefIgArd trial, our work complements it by providing effectiveness data in high-risk patients frequently encountered in routine practice but poorly represented in randomized trials.

The greater benefit of Nefecon over RASi observed across disease severity strata aligns with its mechanism of action targeting gut-associated lymphoid tissue to reduce pathogenic Gd-IgA1 production, the initiating step in IgAN pathogenesis (9, 18). Heavier proteinuria at baseline predicted larger absolute reductions with Nefecon, likely reflecting suppression of the primary immune driver and consequent attenuation of glomerular immune complex deposition and inflammatory injury (26). Conversely, more pronounced eGFR preservation in patients with advanced kidney disease suggests that even late-stage functional decline remains partially modifiable through immunologic intervention, challenging conventional views on disease progression in this population (6). These efficacy patterns across high-risk subgroups, together with a manageable safety profile, support the positioning of Nefecon in the evolving treatment algorithm for IgAN, consistent with the latest KDIGO recommendations (9).

A key real-world practice pattern in our cohort was the concomitant use of immunosuppressants with Nefecon. This combination approach, prohibited in the NeflgArd trial, reflects current clinical practice in China. Recent real-world studies have similarly reported combination therapy in 20% to 35% of Nefecon-treated patients, with some suggesting additive antiproteinuric benefit (21, 22). Subgroup analyses in our cohort did not suggest that concomitant immunosuppressant use substantially modified the treatment effect. Nonetheless, the differences in entry criteria and concomitant therapy mean that our findings should be viewed as complementary to, rather than confirmatory for the RCT results. A notable finding of this study was the high rate of delayed treatment response to Nefecon. More than half of patients classified as non-responders at 1 month ultimately achieved remission by 9 months. This observation carries significant clinical implications: premature treatment discontinuation based on early assessment would have deprived 48.9% of eventual responders of therapeutic benefit. The durability of early partial responses and the progressive 4.6-fold increase in complete remission rates suggest that Nefecon induces sustained, cumulative immunomodulatory effect.

Keskinis C et al. reported a study analyzing the factors influencing the early response to Nefecon treatment in IgAN. They found that patients who demonstrated a very early response had a shorter time interval since diagnosis (27). They also proposed that repeat kidney biopsy can play a vital role in the management of IgAN patients, particularly in those with older diagnoses, as it helps distinguish between the presence of immunological activity and advanced CKD (28). In our patients, the correlation between the interval from kidney biopsy to Nefecon initiation and treatment response was also analyzed. Patients with a time interval of less than 4 years showed a significantly earlier reduction in proteinuria.

The adverse event profile mirrored known corticosteroid effects. Commonly reported adverse events in the NefIgArd trial included hypertension, peripheral edema, and glucocorticoid-related symptoms such as acne, weight gain, and facial edema (10, 11, 23). In our real-world cohort, menstrual disturbance emerged as the most frequent complaint among women of childbearing age. Ouyang et al. reported 11 Chinese patients with IgAN treated with Nefecon, where two of four female patients developed menstrual disorders, which improved after dosage adjustment (29). Similarly, in a Greek cohort of 37 patients, one of 11 female participants experienced menstrual irregularities (27).These findings are mechanistically consistent with the known suppressive effect of corticosteroids on the hypothalamic–pituitary–ovarian axis (30). Although prior reports have linked menstrual disorders to triamcinolone injections (31, 32), whether Nefecon produces comparable effects through local or systemic pathways requires further exploration. This finding suggests the importance of counseling and monitoring reproductive-age women receiving Nefecon.

Several limitations should be acknowledged. First, the retrospective observational design and non-randomized treatment assignment carry inherent risks of confounding. Clinicians preferentially prescribed Nefecon to patients with more severe disease, including lower eGFR and advanced histologic damage, creating channeling bias that likely underestimates true treatment effects, since the Nefecon group faced higher baseline progression risk. Although matching achieved good balance for most covariates, the possibility of unmeasured confounding cannot be excluded. Second, regression to the mean could partially explain the observed proteinuria declines, yet the gradual, sustained improvement over 9 months argues against this as the primary explanation. The reduction magnitude exceeded typical regression artifacts, and results remained consistent across unmatched and matched analyses. Third, the 9-month follow-up precludes evaluation of long-term kidney survival or ESKD incidence. Extended follow-up studies are needed to confirm whether these real-world benefits translate into durable kidney protection and hard endpoint reduction.

## 5 Conclusion

This real-world study found that Nefecon use was associated with greater proteinuria reductions and better kidney function preservation in patients with IgAN, including those with advanced disease. Patients with higher baseline proteinuria experienced greater reductions in proteinuria, while those with lower eGFR showed more pronounced kidney function preservation. These patterns may help guide treatment decisions in clinical practice. Menstrual irregularities were common among female patients, highlighting the importance of discussing this potential side effect and monitoring accordingly. These findings warrant confirmation in prospective, long-term studies.

## Data Availability

Access can be granted upon reasonable request to the corresponding authors.
