# Supplementary material for: Real-world multicenter evidence on Nefecon for attenuating proteinuria and kidney function decline in IgA nephropathy
Source: Front Immunol. 2026 Aug 18;17:1860166. doi: 10.3389/fimmu.2026.1860166 (PMC13529767; doi:10.3389/fimmu.2026.1860166)
Supplement: Supplementary file 1 [file Table1.pdf]

Table S1. Baseline Characteristics of Mahalanobis-Matched RASi and Nefecon groups (n = 80).

Variables	Total (n=80)	RASi group (n=40)	Nefecon group (n=40)	*P*	SMD
Age, years	37.8 ± 9.3	38.1 ± 9.8	37.5 ± 9.0	0.800	0.056
Female sex, n (%)	42 (52.5%)	21 (52.5%)	21 (52.5%)	1.000	<0.001
Total follow-up duration, months	53.9 ± 57.3	64.2 ± 58.3	43.6 ± 54.9	0.054	0.362
BMI, Kg/m²	23.0 ± 4.2	22.6 ± 4.4	23.4 ± 3.9	0.937	0.178
SBP, mmHg	126.0 ± 26.2	124.4 ± 29.9	127.9 ± 21.4	0.820	0.133
DBP, mmHg	83.3 ± 15.0	83.2 ± 10.6	83.5 ± 19.2	0.909	0.021
Hypertension, n (%)	16 (32.0%)	13 (32.5%)	3 (30.0%)	1.000	0.052
DM, n (%)	2 (2.5%)	1 (2.5%)	1 (2.5%)	1.000	<0.001
Proteinuria, g/24h	1.6 ± 1.0	1.6 ± 0.7	1.6 ± 1.3	0.279	0.009
SCr, umol/L	123.2 ± 57.1	122.6 ± 67.1	123.7 ± 45.9	0.211	0.020
eGFR, ml/min/1.73m²	67.4 ± 26.6	68.5 ± 29.2	66.4 ± 24.1	0.747	0.077
UA, umol/L	388.7 ± 94.6	398.6 ± 104.2	377.8 ± 82.5	0.340	0.221
Serum albumin, g/L	41.6 ± 3.7	41.7 ± 4.0	41.4 ± 3.3	0.710	0.088
SGLT2i, n (%)	42 (52.5%)	20 (50.0%)	22 (55.0%)	0.823	0.100
MEST-C score, n (%)					
M1	51 (69.9%)	27 (69.2%)	24 (70.6%)	1.000	0.156
E1	21 (28.8%)	11 (28.2%)	10 (29.4%)	1.000	0.057
S1	57 (78.1%)	29 (74.4%)	28 (82.4%)	0.590	0.055
T1	15 (20.5%)	7 (17.9%)	8 (23.5%)	0.770	0.057
T2	6 (8.2%)	3 (7.7%)	3 (8.8%)	1.000	<0.001
C1	16 (22.9%)	7 (18.9%)	9 (27.3%)	0.590	0.125
C2	0	0	0	-	-

Propensity Score Matching (1:1, caliper=0.02) was performed using age, sex, eGFR, proteinuria, serum albumin, SGLT2i use, and Oxford MEST-C classification scores as covariates.

SMD, Standardized Mean Difference; BMI, body mass index; SBP, Systolic Blood Pressure；DBP, Diastolic Blood Pressure; SCr, Serum creatinine; eGFR, estimated glomerular filtration rate; UA, uric acid; SGLT2i, Sodium-Glucose Cotransporter 2 Inhibitor. M, mesangial hypercellularity (M0, <50% of glomeruli show mesangial hypercellularity; M1, >50% of glomeruli show mesangial hypercellularity); E, endocapillary hypercellularity (E0, no endocapillary hypercellularity; E1, any glomeruli show endocapillary hypercellularity); S, segmental glomerulosclerosis (S0, absent; S1, present in any glomeruli); T, tubular atrophy/interstitial fibrosis (T0, 0%-25% of cortical area; T1, 26%-50% of cortical area; T2, >50% of cortical area); C, crescents (C0: absent; C1, 0%-25% of glomeruli; C2, ≥25% of glomeruli).

Table S2. Time-point-specific missing data rates for key variables in Nefecon group

Time point (month)	M0	M1	M2	M3	M4	M5	M6	M7	M8	M9
Proteinuria (g/24h)	0	10 (12.7%)	11 (13.9%)	11 (13.9%)	15 (19.0%)	14 (17.7%)	13 (16.5%)	19 (24.1%)	16 (20.3%)	2 (2.5%)
eGFR(ml/min/1.73m²)	0	4 (5.1%)	4 (5.1%)	4 (5.1%)	5 (6.3%)	11 (13.9%)	8 (10.1%)	11 (13.9%)	13 (16.5%)	3 (3.8%)

Table S3. Treatment Adherence and Dose Adjustments.

Parameter	Nefecon group (n=79)
Completed full 9-month course, n (%)	79 (100%)
Dose reduction or temporary interruption, n (%)	4 (5.1%)
— Adverse events	2 (2.5%)
— Patient decision	1 (1.3%)
— Economic reasons	1 (1.3%)
Timing of dose adjustment, n (%)	
— Month 1–3	1 (1.3%)
— Month 4–6	1 (1.3%)
— Month 7–9	2 (2.5%)

Note: Temporary dose reduction: Nefecon reduced from 16 mg to 8 mg daily. All enrolled patients completed the full 9-month course per retrospective cohort design; patients with premature discontinuation were excluded at enrollment (see STROBE flow diagram, Figure S1).

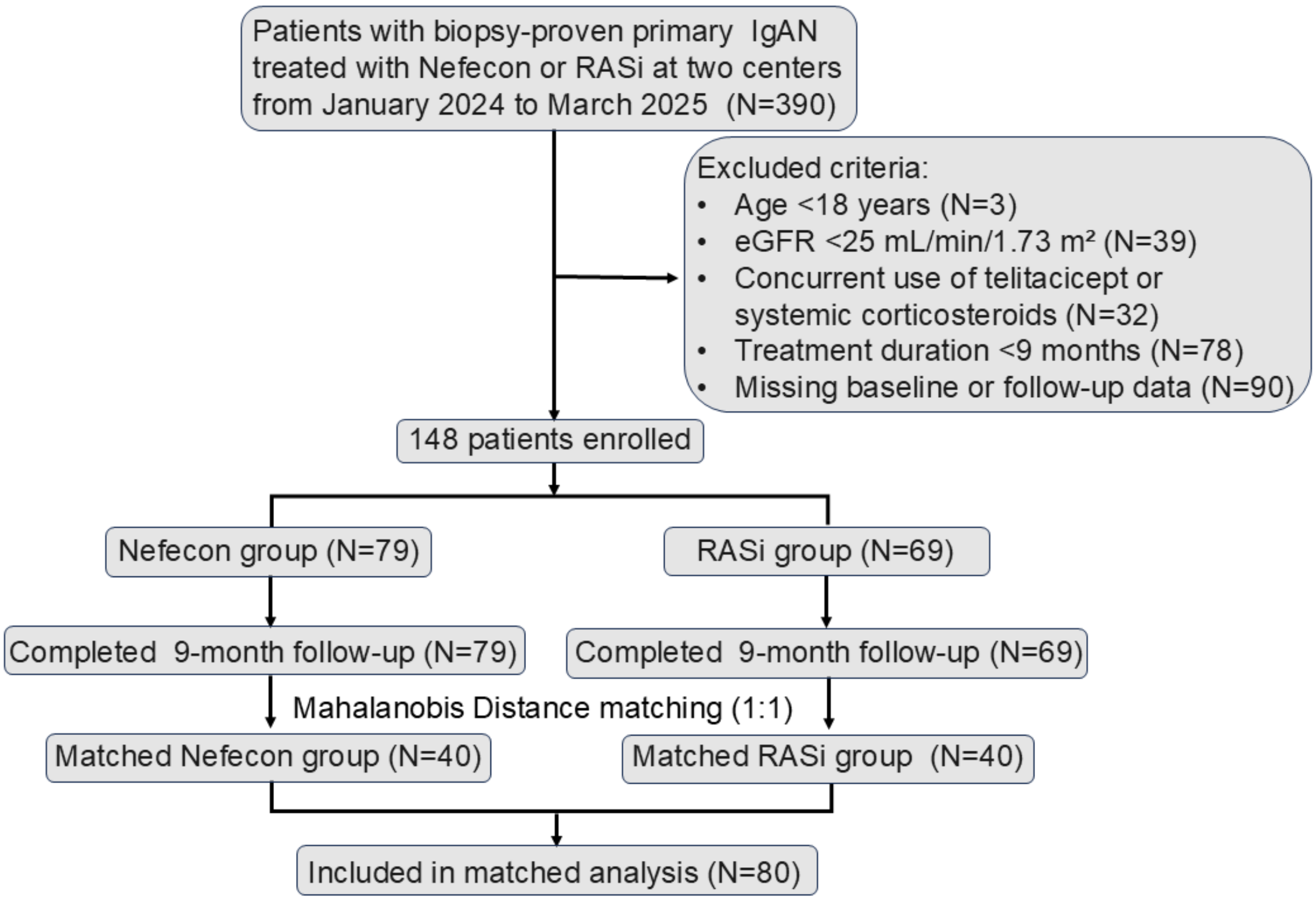

Figure S1. STROBE-Compliant participant flow diagram.

A total of 390 patients with biopsy-proven primary IgAN were screened at two centers between January 2024 and March 2025. After excluding 242 patients, 148 were enrolled and divided into the Nefecon group (n=79) and the RASi group (n=69). All enrolled patients completed the 9-month follow-up. Mahalanobis Distance matching (1:1, caliper=0.02) was performed using age, sex, eGFR, proteinuria, serum albumin, SGLT2i use, and Oxford MEST-C classification scores as covariates, yielding 40 matched pairs for the primary analysis. RASi, renin-angiotensin system inhibitor.

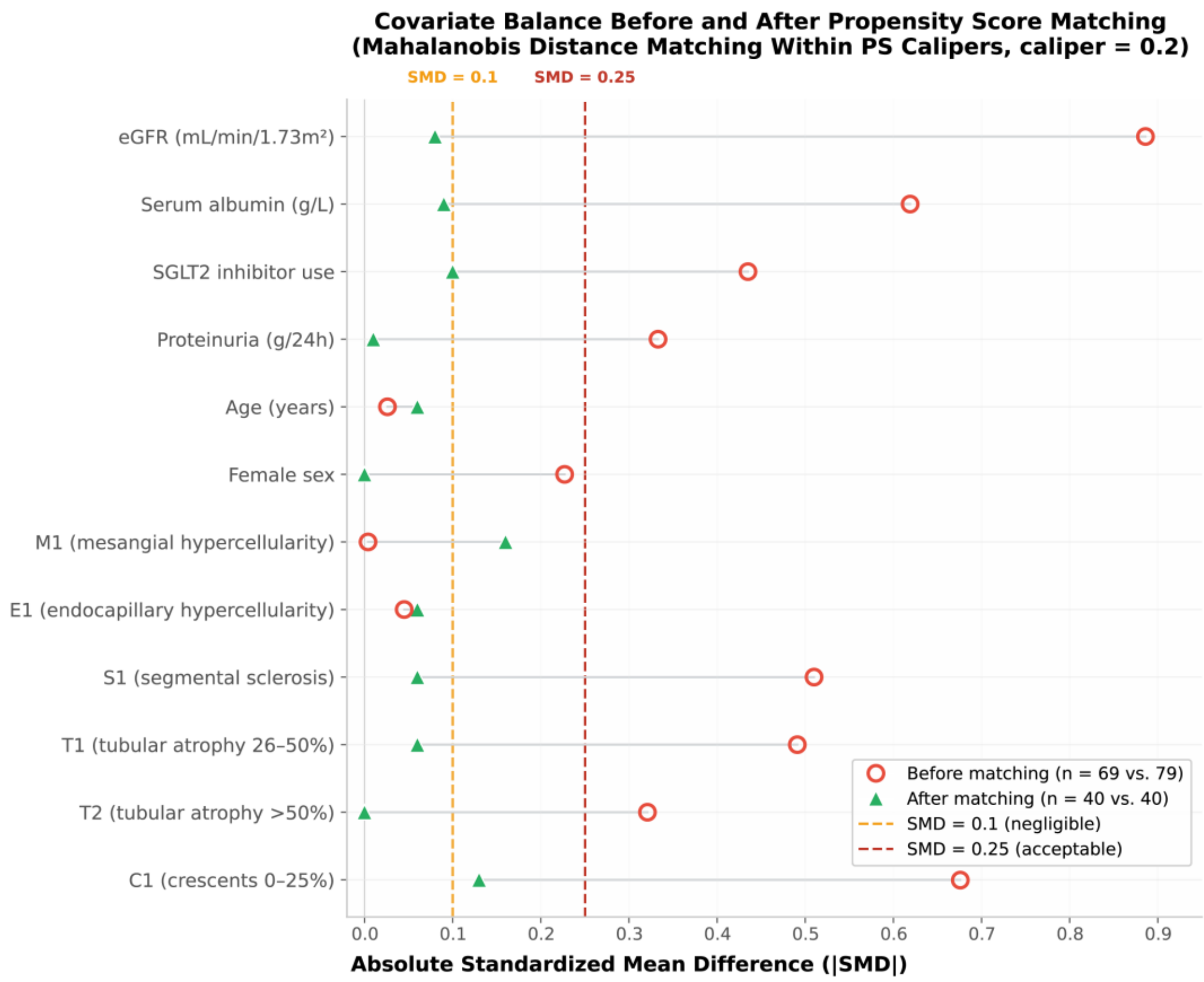

Figure S2. Love Plot of standardized mean differences (SMDs) for baseline covariates before and after Mahalanobis distance matching within Propensity Score Calipers.

Mahalanobis distance matching within propensity score calipers (caliper = 0.2 standard deviations of the logit propensity score) was performed using a 1:1 nearest-neighbor algorithm without replacement on 12 baseline covariates: age, sex, eGFR, proteinuria, serum albumin, SGLT2 inhibitor use, and five Oxford MEST-C components (M1, E1, S1, T1, T2, C1). Open red circles represent absolute SMDs before matching (RASi n = 69; Nefecon n = 79). Filled green triangles represent absolute SMDs after matching (RASi n = 40; Nefecon n = 40). Gray horizontal lines connect before and after values for each covariate. The dashed orange line indicates SMD = 0.1 (negligible imbalance); the dashed red line indicates SMD = 0.25 (acceptable balance threshold). All 12 matching covariates achieved SMD < 0.2 after matching; 9 of 12 achieved SMD < 0.1.

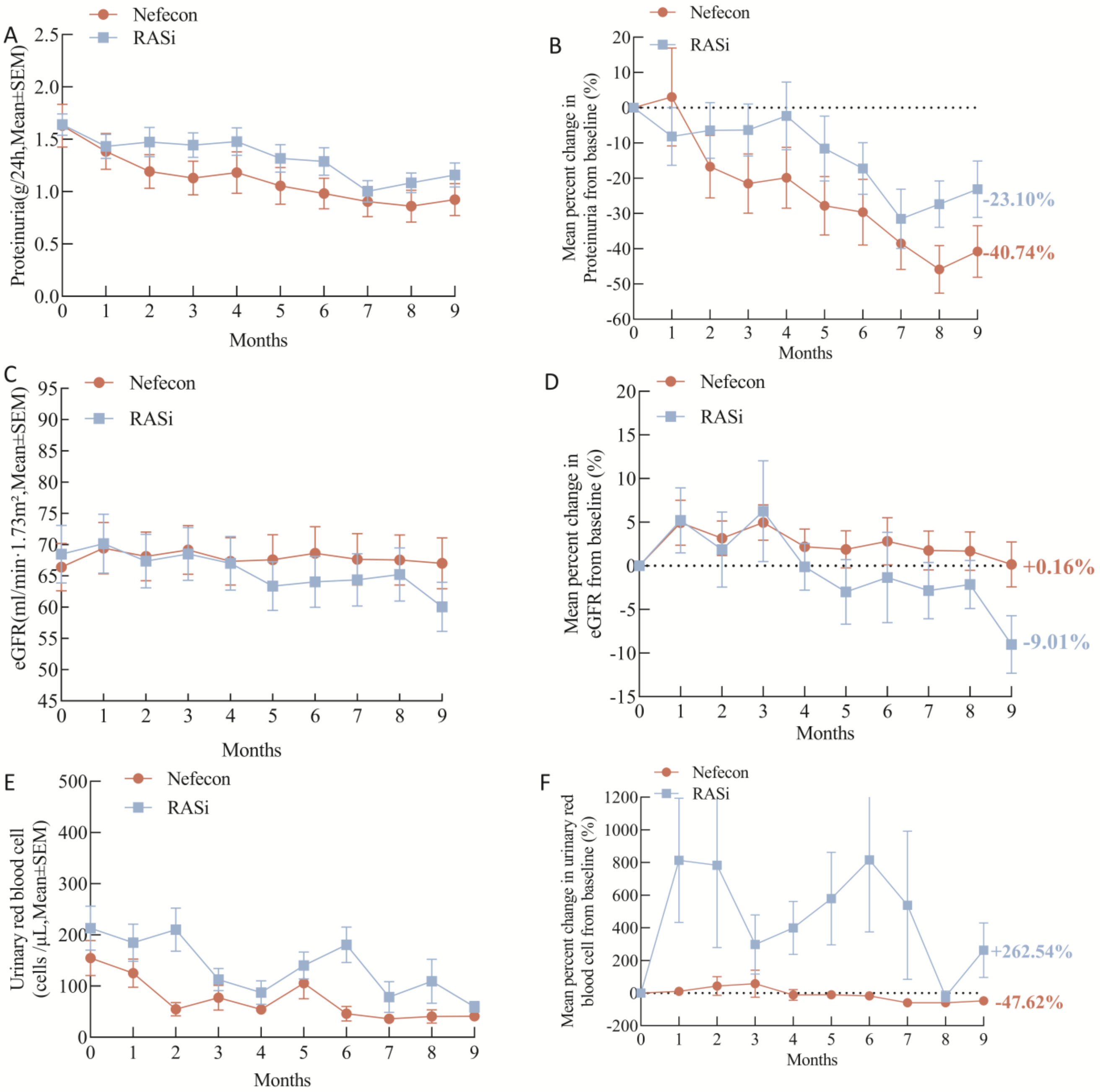

Figure S3. Changes in clinical parameters from baseline to 9 months after Propensity Score Matching.

A. Proteinuria (g/24h), C. eGFR (mL/min/1.73 m^2), and E. urinary red blood (cells/μl) from baseline to 9 months in RASi and Nefecon groups.

Mean percent change in B. Proteinuria (g/24h), D. eGFR (mL/min/1.73 m^2), and F. urinary red blood cells (cells/μl) from baseline to 9 months. Data are presented as mean ± standard error.

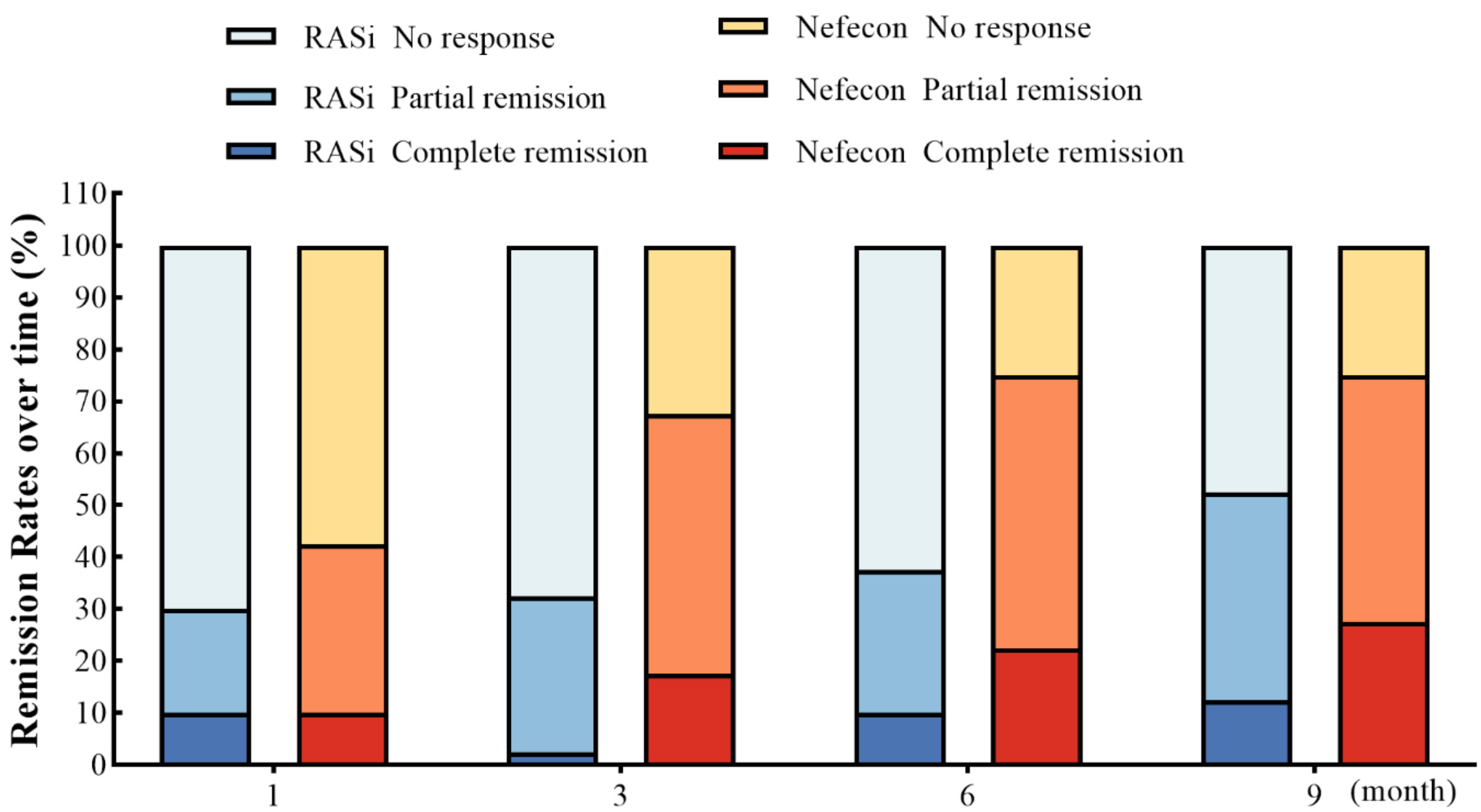

	RASi	Nefecon	*P*	RASi	Nefecon	*P*	RASi	Nefecon	*P*	RASi	Nefecon	*P*
No response	70.0%	57.5%		67.5%	32.5%		62.5%	25.0%		47.5%	25.0%	
Partial remission	20.0%	32.5%	0.432	30.0%	50.0%	0.003	27.5%	52.5%	0.003	40.0%	47.5%	0.071
Complete remission	10.0%	10.0%		2.5%	17.5%		10.0%	22.5%		12.5%	27.5%	

Figure S4. Remission rates between treatment groups over time after Propensity Score Matching. Stacked bars show the proportion of patients achieving complete remission, partial remission, or no response at 1, 3, 6, and 9 months after treatment. The Nefecon group demonstrated significantly higher cumulative remission rates (CR+PR) compared to the RASi group.

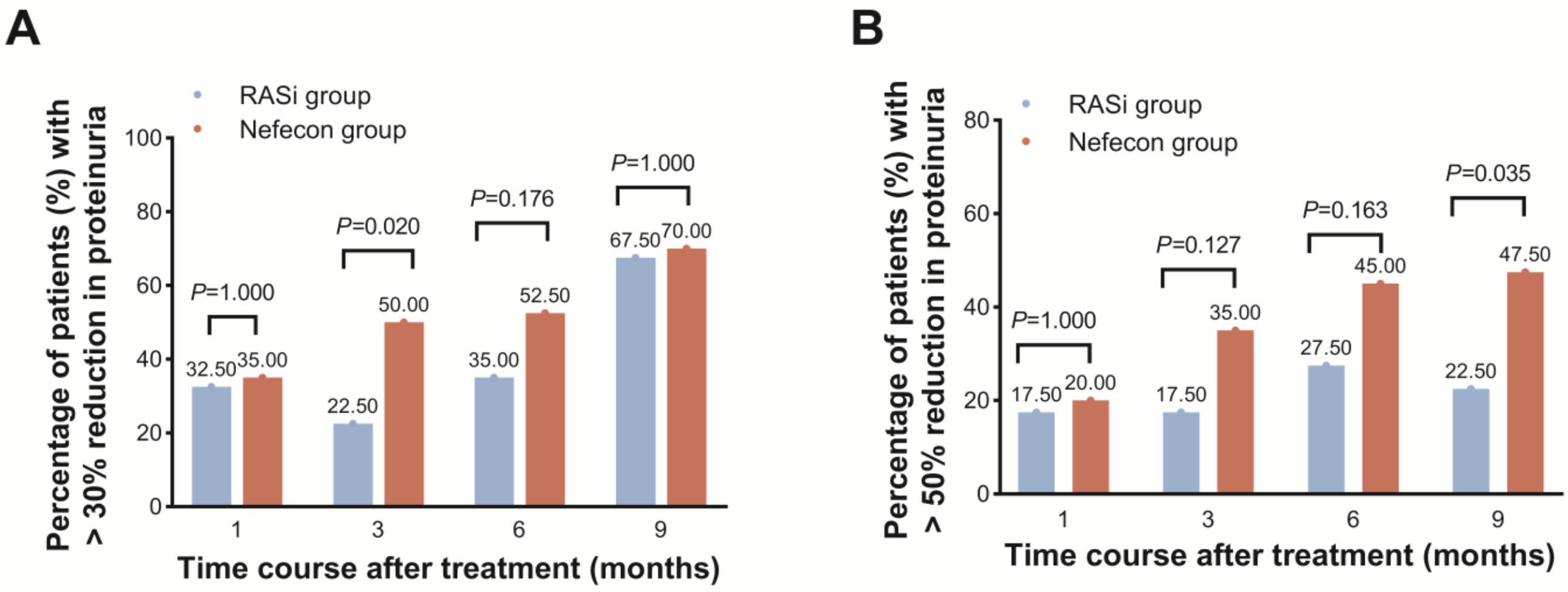

Figure S5. Proportion of patients with ≥30% (A) or ≥50% (B) reduction in 24-hour proteinuria from baseline at 1, 3, 6 and 9 months after Propensity Score Matching.

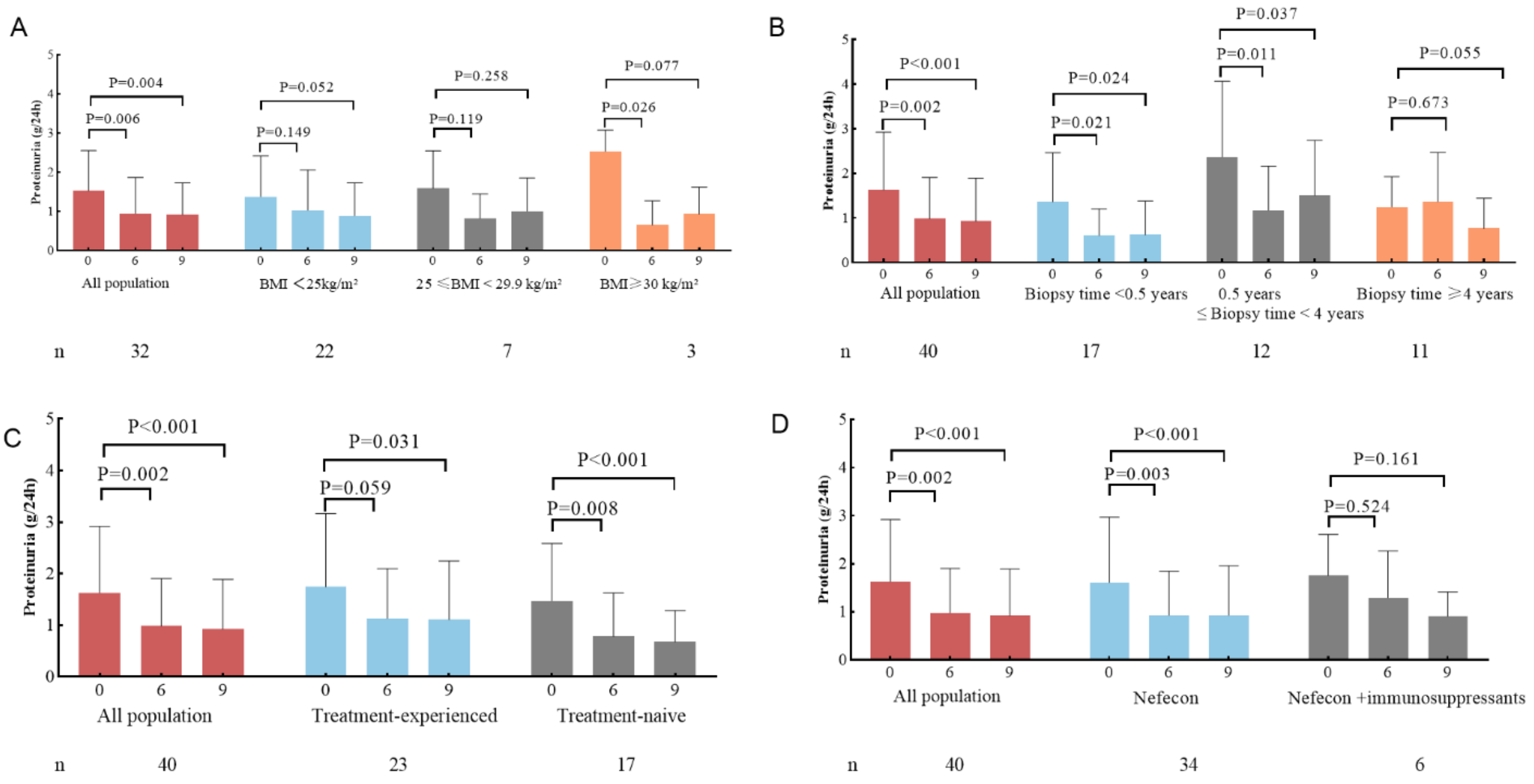

Figure S6. Proteinuria reduction with Nefecon across patient subgroups after Propensity Score Matching.

Bar graph showing the mean absolute reduction in proteinuria from baseline to 9 months in the Nefecon group, stratified by the following subgroups: (A) baseline BMI, (B) biopsy time, (C)treatment-naive versus treatment-experienced patients, (D) Nefecon monotherapy versus Nefecon combined with immunosuppressants.

Note:

Treatment-naive: patients who had never received any prior systemic therapy (including corticosteroids, immunosuppressants, or biologics) before study entry. Treatment-experienced: patients who had previously received one or more systemic therapies for their underlying disease, regardless of duration or response.

Nefecon monotherapy: patients received Nefecon alone without concomitant immunosuppressive agents.

Nefecon + immunosuppressants: patients received Nefecon along with any systemic immunosuppressive medications (e.g., azathioprine, mycophenolate, calcineurin inhibitors) during the study period.

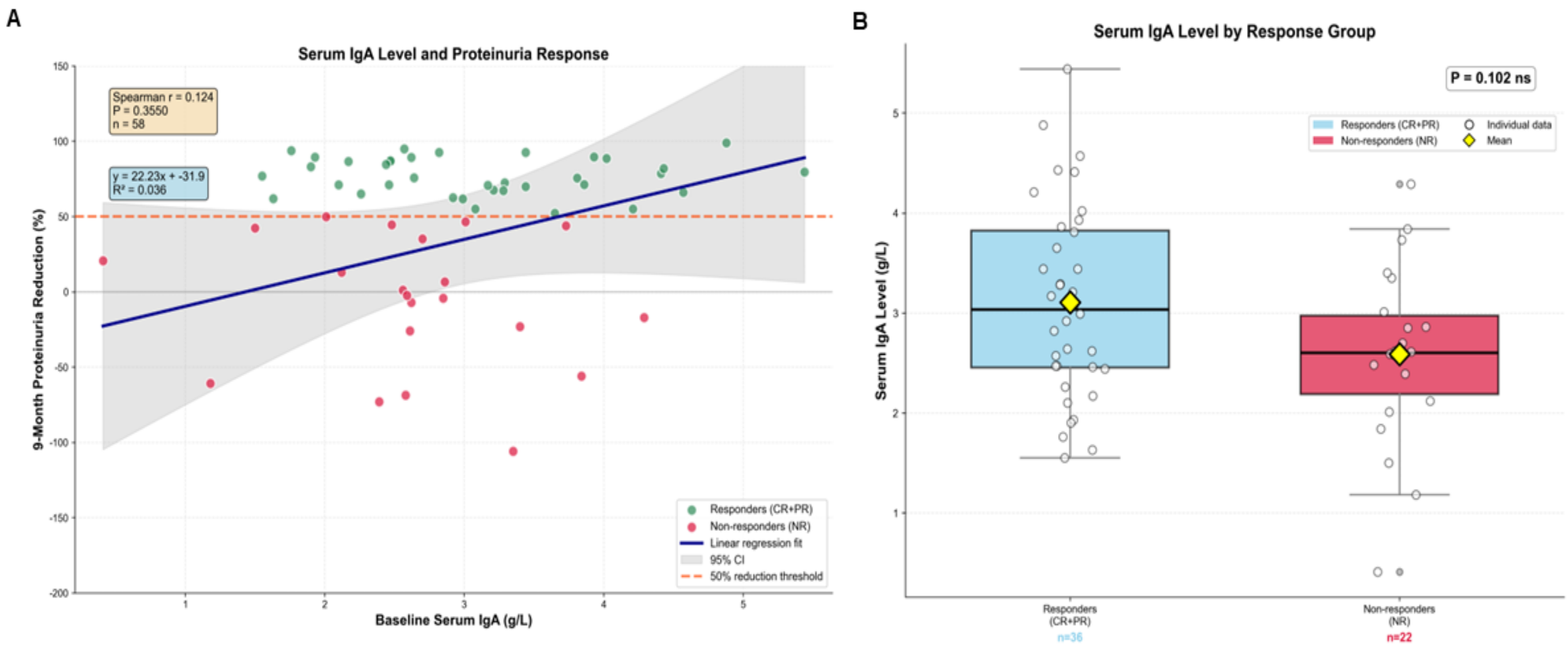

Figure S7. Baseline serum IgA levels and treatment response to Nefecon.

(A) Scatter plot showing the relationship between baseline serum IgA levels (g/L) and 9-month proteinuria reduction (%). Spearman correlation analysis (r = 0.124, P = 0.3550, n = 58) and linear regression (blue line, $y = 22.23x - 31.9$, $R^2 = 0.036$) are shown, with the 95% confidence interval (gray shaded area) and 50% proteinuria reduction threshold (orange dashed line) indicated. Responders (CR+PR) are represented by green dots, and non-responders (NR) by red dots.

(B) Box plot of baseline serum IgA levels (g/L) stratified by treatment response. The box represents the interquartile range, the black line represents the median, and the yellow diamond represents the mean. Individual data points are shown as open circles. No significant difference in baseline IgA levels was observed between responders (n = 36) and non-responders (n = 22; *P* = 0.102).

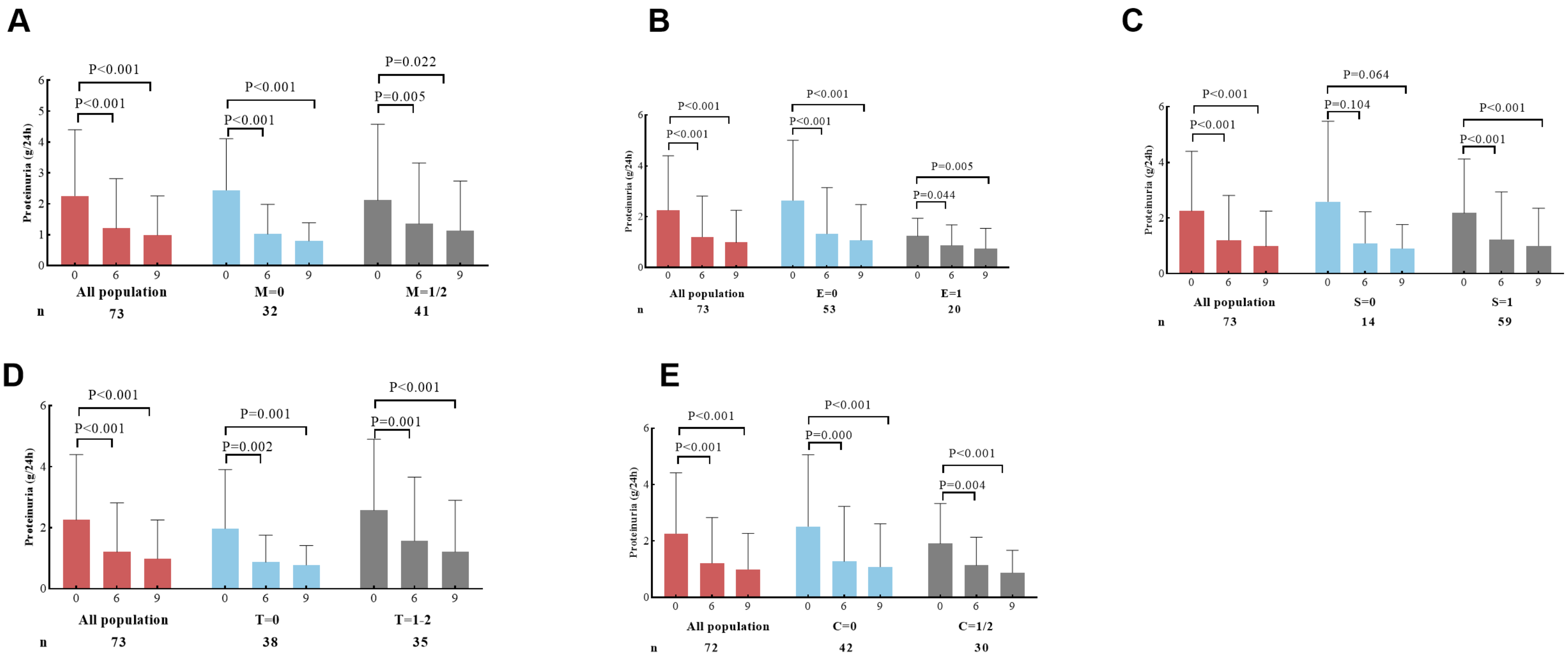

Figure S8. Subgroup analysis of proteinuria changes from baseline to Month 9 in Nefecon-treated patients stratified by MEST-C score components.

P-values represent within-group comparisons. All interaction P-values > 0.05.

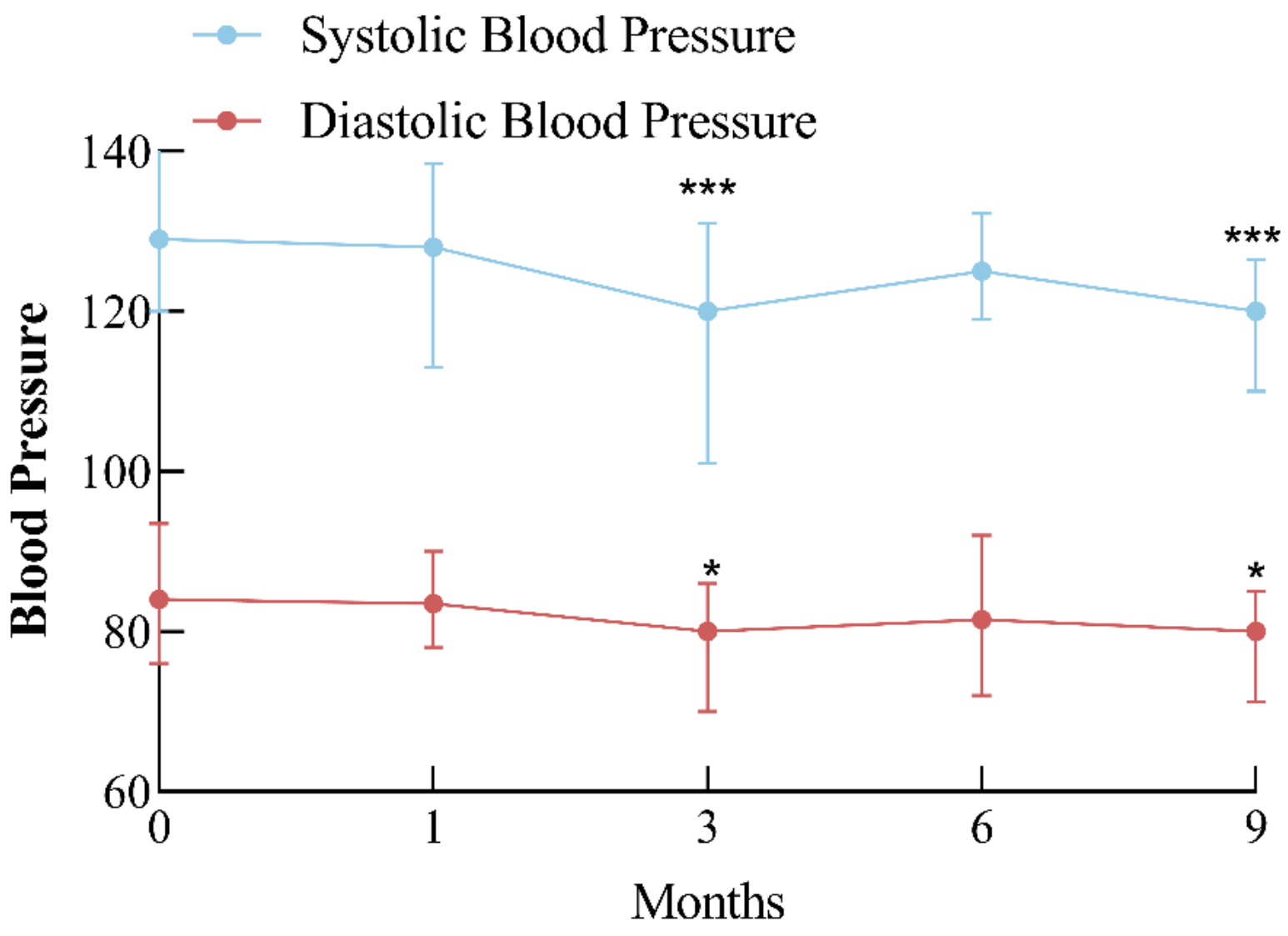

Figure S9. Serial measurements of systolic and diastolic blood pressure at baseline, 3, 6, and 9 months in Nefecon-treated patients

P-values were calculated by comparing blood pressure at each follow-up time point with baseline values within the Nefecon group (n=79). Asterisks indicate levels of statistical significance: *P < 0.05; **P < 0.01; ***P < 0.001.

STROBE Statement—checklist of items that should be included in reports of observational studies

	Item No	Recommendation	Page No
**Title and abstract**	1	(*a*) Indicate the study's design with a commonly used term in the title or the abstract	1
		(*b*) Provide in the abstract an informative and balanced summary of what was done and what was found	4
**Introduction**			
Background/rationale	2	Explain the scientific background and rationale for the investigation being reported	6
Objectives	3	State specific objectives, including any prespecified hypotheses	7
**Methods**			
Study design	4	Present key elements of study design early in the paper	7
Setting	5	Describe the setting, locations, and relevant dates, including periods of recruitment, exposure, follow-up, and data collection	7
Participants	6	(*a*) *Cohort study*—Give the eligibility criteria, and the sources and methods of selection of participants. Describe methods of follow-up<br>*Case-control study*—Give the eligibility criteria, and the sources and methods of case ascertainment and control selection. Give the rationale for the choice of cases and controls<br>*Cross-sectional study*—Give the eligibility criteria, and the sources and methods of selection of participants	7
		(*b*) *Cohort study*—For matched studies, give matching criteria and number of exposed and unexposed<br>*Case-control study*—For matched studies, give matching criteria and the number of controls per case	
Variables	7	Clearly define all outcomes, exposures, predictors, potential confounders, and effect modifiers. Give diagnostic criteria, if applicable	8
Data sources/ measurement	8*	For each variable of interest, give sources of data and details of methods of assessment (measurement). Describe comparability of assessment methods if there is more than one group	*8-9*
Bias	9	Describe any efforts to address potential sources of bias	
Study size	10	Explain how the study size was arrived at	
Quantitative variables	11	Explain how quantitative variables were handled in the analyses. If applicable, describe which groupings were chosen and why	8-9
Statistical methods	12	(*a*) Describe all statistical methods, including those used to control for confounding	8-9
		(*b*) Describe any methods used to examine subgroups and interactions	
		(*c*) Explain how missing data were addressed	
		(*d*) *Cohort study*—If applicable, explain how loss to follow-up was addressed<br>*Case-control study*—If applicable, explain how matching of cases and controls was addressed<br>*Cross-sectional study*—If applicable, describe analytical methods taking account of sampling strategy	
		(*e*) Describe any sensitivity analyses	

Continued on next page

**Results**			
Participants	13*	(a) Report numbers of individuals at each stage of study—eg numbers potentially eligible, examined for eligibility, confirmed eligible, included in the study, completing follow-up, and analysed	9
		(b) Give reasons for non-participation at each stage	
		(c) Consider use of a flow diagram	
Descriptive data	14*	(a) Give characteristics of study participants (eg demographic, clinical, social) and information on exposures and potential confounders	9
		(b) Indicate number of participants with missing data for each variable of interest	
		(c) *Cohort study*—Summarise follow-up time (eg, average and total amount)	
Outcome data	15*	*Cohort study*—Report numbers of outcome events or summary measures over time	*9-13*
		Case-control study—Report numbers in each exposure category, or summary measures of exposure	
		Cross-sectional study—Report numbers of outcome events or summary measures	
Main results	16	(*a*) Give unadjusted estimates and, if applicable, confounder-adjusted estimates and their precision (eg, 95% confidence interval). Make clear which confounders were adjusted for and why they were included	9-13
		(*b*) Report category boundaries when continuous variables were categorized	
		(*c*) If relevant, consider translating estimates of relative risk into absolute risk for a meaningful time period	
Other analyses	17	Report other analyses done—eg analyses of subgroups and interactions, and sensitivity analyses	
**Discussion**			
Key results	18	Summarise key results with reference to study objectives	13-14
Limitations	19	Discuss limitations of the study, taking into account sources of potential bias or imprecision. Discuss both direction and magnitude of any potential bias	16
Interpretation	20	Give a cautious overall interpretation of results considering objectives, limitations, multiplicity of analyses, results from similar studies, and other relevant evidence	13
Generalisability	21	Discuss the generalisability (external validity) of the study results	16
**Other information**			
Funding	22	Give the source of funding and the role of the funders for the present study and, if applicable, for the original study on which the present article is based	17

*Give information separately for cases and controls in case-control studies and, if applicable, for exposed and unexposed groups in cohort and cross-sectional studies.

Note: An Explanation and Elaboration article discusses each checklist item and gives methodological background and published examples of transparent reporting. The STROBE checklist is best used in conjunction with this article (freely available on the Web sites of PLoS Medicine at http://www.plosmedicine.org/, Annals of Internal Medicine at http://www.annals.org/, and Epidemiology at http://www.epidem.com/). Information on the STROBE Initiative is available at www.strobe-statement.org.
